# Ensemble Modeling Approach Targeting Heterogeneous RNA-Seq data: Application to Melanoma Pseudogenes

**DOI:** 10.1038/s41598-017-17337-7

**Published:** 2017-12-11

**Authors:** Enrico Capobianco, Camilo Valdes, Samanta Sarti, Zhijie Jiang, Laura Poliseno, Nicolas F. Tsinoremas

**Affiliations:** 1 Center for Computational Science, University of Miami, Miami, FL USA; 2 University of Siena, Siena, Italy; 3 Istituto Toscano Tumori Oncogenomics Unit, Institute of Clinical Physiology-National Research Council, Pisa, Italy; 4 Department of Medicine, Miller School of Medicine, University of Miami, Miami, FL USA

## Abstract

We studied the transcriptome landscape of skin cutaneous melanoma (SKCM) using 103 primary tumor samples from TCGA, and measured the expression levels of both protein coding genes and non-coding RNAs (ncRNAs). In particular, we emphasized pseudogenes potentially relevant to this cancer. While cataloguing the profiles based on the known biotypes, all the employed RNA-Seq methods generated just a small consensus of significant biotypes. We thus designed an approach to reconcile the profiles from all methods following a simple strategy: we selected genes that were confirmed as differentially expressed by the ensemble predictions obtained in a regression model. The main advantages of this approach are: 1) Selection of a high-confidence gene set identifying relevant pathways; 2) Use of a regression model whose covariates embed all method-driven outcomes to predict an averaged profile; 3) Method-specific assessment of prediction power and significance. Furthermore, the approach can be generalized to any biological system for which noisy RNA-Seq profiles are computed. As our analyses concerned bio-annotations of both high-quality protein coding genes and ncRNAs, we considered the associations between pseudogenes and parental genes (targets). Among the candidate targets that were validated, we identified PINK1, which is studied in patients with Parkinson and cancer (especially melanoma).

## Introduction

Melanoma, the deadliest of skin cancers, is a malignant and highly metastatic tumor with growing incidence of about 20 per 100,000 individuals in Western countries^1^. In 10% of melanoma cases, there is a positive family history, i.e. a risk factor significantly contributing to disease occurrence^2^. At a genetic level, multiple genes are naturally expected to interactively exert direct and/or indirect influences on many biological processes and pathways, suggesting possible signatures of the disease. One of the first discovery efforts in melanoma transcriptome studies revealed the emergence of genomic rearrangements behind novel gene fusions and identified somatic mutations responsible for disease progression^3^. Further RNA-Seq analyses have provided in-depth screening for a variety of transcript structures, by studying human samples^4^ or cell lines^5^. RNA-Seq is the standard technique that is used to characterize cancer transcriptomes while measuring known transcripts and predicting novel transcripts^6–11^. Additionally, RNA-Seq expands the detection power of transcriptome profiles by identifying and elucidating a variety of non-coding RNAs (ncRNAs)^12,13^. Following the seminal findings of two large-scale projects, ENCODE^14^ (https://www.encodeproject.org/) and GENCODE^15^ (http://www.gencodegenes.org/), the general attention has been gradually diverted away from genes and concentrated over other biotypes, such as long intergenic ncRNAs (lincRNAs), antisense RNAs, pseudogenes etc. In most cases the identification of potentially transcribed bio-entitities was inferred from locus-specific (EST) and wide-spectrum experiments, then referring transcriptional activity to tissue specificity and establishing association with active chromatin state and promoter regions.

We performed a systematic exploration of the skin cutaneous melanoma (SKCM) transcriptome landscape by accessing the TCGA repository (http://cancergenome.nih.gov/) and its entire collection of 103 primary SKCM samples. As TCGA contained only one control sample for SKCM, we retrieved other reference melanocyte samples in the GEO repository (http://www.ncbi.nlm.nih.gov/geo/). In order to identify both gene and ncRNA signatures, we built an ad hoc methodological pipeline to deal with the observed biotype variety and expression heterogeneity. Pervasive transcription has been widely documented, involving multi-exonic transcripts that map to intergenic regions with specific interactions and functions^16–22^. The ability to detect ncRNAs suffers from low read density coverage, thereby resulting in non-uniform coverage of transcript abundance. This latter is generally >100-fold higher in exons than in introns or intergenic regions. However, despite the limitation of more/less abundant transcripts represented by many/few reads, respectively, lowly expressed transcripts can be widely detected by RNA-Seq across many cancers.

The pseudogene participation to the transcriptional activity deserves a special attention. Thousands of pseudogenes have been identified in the human genome, many with critical regulatory functions^23^. For instance, an estimate indicates 12683 human pseudogenes, with 11216 surveyed as consensus pseudogenes and 9% actively transcribed. Since pseudogenes lack protein coding potential, they can manifest functional or non-functional RNA products, and appear specific with respect to both tissues and cells. ENCODE has identified about 15000 pseudogenes, a great majority of which are transcriptionally active. A recent large-scale analysis of pseudogene transcription in 13 cancer types (248 cancer samples, and 45 normal samples) showed the signature of 2082 distinct pseudogenes^4^. Several aspects of pseudogenes involve uncertainty with regard to identification, function, annotation, role in cancer^24–26^. We believe that knowledge gains can be derived from the use of computational approaches^27–29^. For example, the associations between pseudogenes and so-called parental genes, i.e. the functional genes with the highest sequence similarity, can be usefully investigated. One reason is that the complexity of such relationships goes far beyond the possible presence of correlation between expression levels. The latter is quite controversial because inconsistently supported by evidence. Based on the analyzed samples, we have provided an empirical assessment of such patterns both at systemic scale and at local level of specific associations^30,31^. Our integrative computational approach involves three main steps, which we applied sequentially to the SKCM transcriptome data.

i) *RNA-Seq analysis*, performed by multiple methods. The results were then integrated to achieve a large consensus of differentially expressed (DE) bioentities, either differentially expressed genes (DEGs) or DE ncRNAs;
ii) *Ensemble statistical modeling*. This step aimed to recover salient features that remain hidden in the consensus data. It reconcileed the profiles obtained from the different methods into a high-confidence inferable coreset of model-classified bioentities;
iii) *Bio-annotations* (pathways and gene families), organized into curated lists of pathway and GO categories. These were supported by other classifications addressing the potential of genes to play a role in cancer (oncogenes, tumor suppressors, etc.).

We performed two complementary methodological steps: a) The identification of pseudogenes and corresponding parental genes, followed by their profiling and by an assessment of their relationships; and b) The functional validation of selected DEG candidates to verify both reliability and effectiveness of our ensemble approach to generate testable hypotheses. The rationale for enabling an ensemble inference approach is that multiple RNA-Seq methods can be used to both quantify and profile the expression levels of transcriptome biotypes, but limitations are present with each method. Clearly enough, studies centered on specific methods are subject to risk of bias. Among the alternative strategies that can be used to overcome the limitations, some establish an efficient practice by combining results from more than one method. It is also possible that contradictory results may emerge from using methods operating different quantifications and normalizations. Our evidences demonstrate exactly this aspect. In particular, a method-driven selection of different and separated DE-space regions is obtained, and the significance is assigned to detections that are in large part not shared. To increase the consistency of the results, evidences should therefore be consolidated without aiming necessarily at a consensus. This goal can be achieved without over-penalizing disparate evidences through the ensemble modeling approach that we introduce here and demonstrate to be reasonably efficient and accurate.

## Results

**Figure 1:**
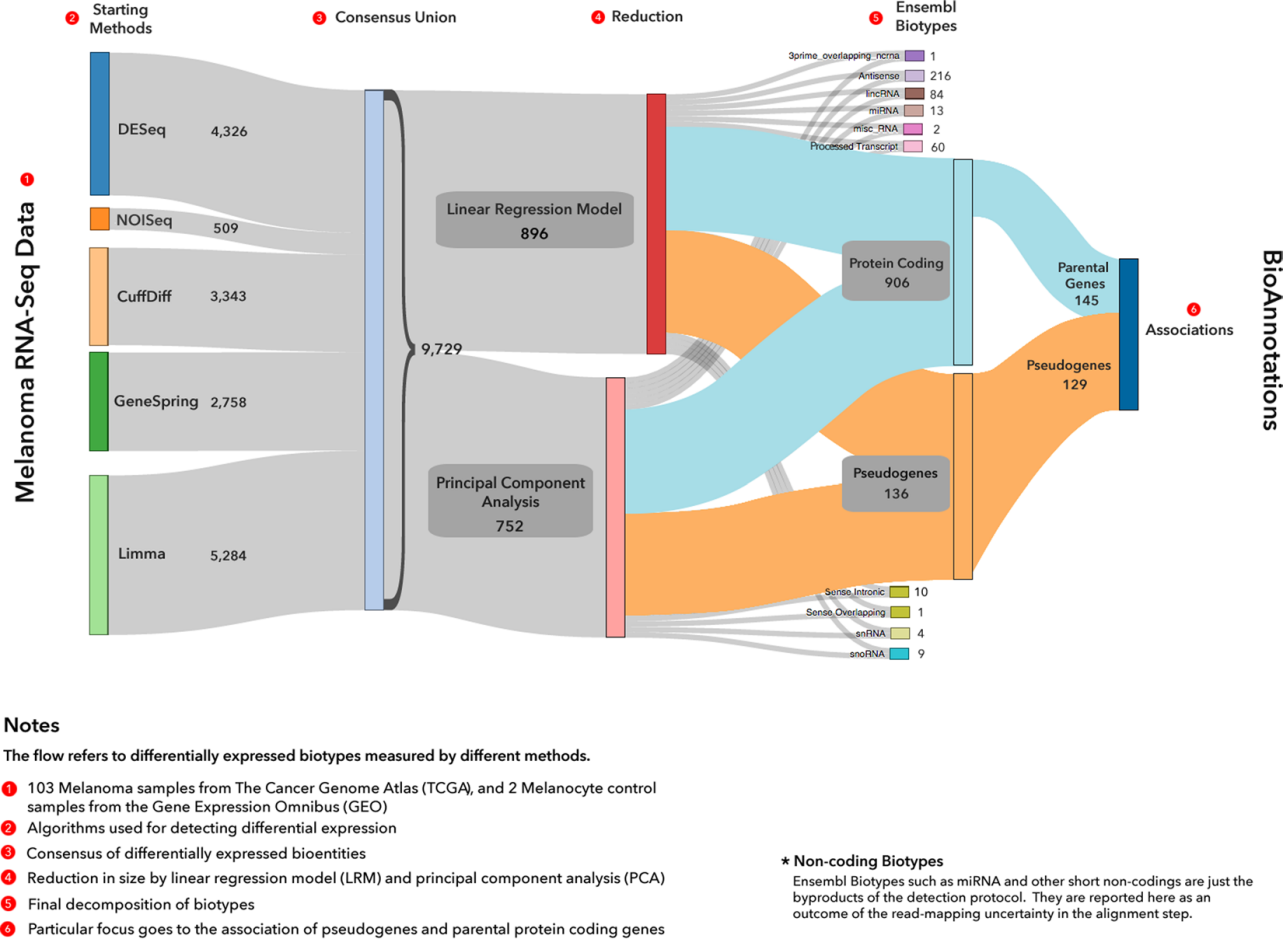
Sankey flow diagram. The outcomes generated by our approach are visualized step-by-step. The first stage indicated at the left side lists the various methods applied to the RNA-Seq data with the corresponding profile, namely the number of DE biotypes (both genes and ncRNAs) detected in each individual application. A total profile of 9,729 DE biotypes is obtained from the sum of all such detections, and in particular Limma (5290) and DESeq (4326) which provided the largest amounts, CuffDiff (3568) and GeneSpring (2758) with smaller amounts, and finally NOISeq as the most conservative methods (511) (Supplementary Tables 5.1.1-5). Two models are then reported, LRM and PCA which deliver respectively 896 and 752 DE biotypes, thus reducing dramatically (more than one order of magnitude) the overall profile. The rest of the diagram accounts for specific biotypes, relatively more represented by protein coding genes (906), antisense (216), lincRNA (84), pseudogenes (136), and with the latter presenting in 129 cases association with related parental genes (145) (Supplementary Fig. 5.2.1). Bioannotations (pathways, gene families) are indicated downstream as the endpoint of the diagram.

Figure 1 concisely illustrates the overall findings of our work through a Sankey flow diagram. The information flow shows the results of applying our methodological pipeline to the TCGA SKCM data. The included steps reflect the numeric evidences found by the application of RNA-Seq methods and model reduction approaches, and the qualitative refinements obtained from biotype catalogues and bioannotations. We can state that modeling through an ensemble approach represents a viable solution to the problem of reconcile multi-evidence RNA-Seq results.

At a biological level our primary interest is to detect the DE profiles of biotypes forming the SKCM transcriptome landscape (Fig. 1, left). Such profiles may be provided by different methods, and we chose five of them to build a consensus. To increase detection accuracy and exclude redundancy, the ensemble modeling was designed to take place after the consensus. The ensemble strategy produces better DE profiling and better biotype decomposition (Fig. 1, right). We look especially at the ncRNAs and focus on pseudogenes and parental genes associations. Statistical models supporting the ensemble are Principal Component Analysis (PCA) and Linear Regression Model (LRM). Their specific structures explain the differential performance. The use of RNA-Seq expression profiles as predictors cast within the LRM framework is central to our inference approach. This way, the profiles generated by the methods are used predictively and their performance assessed synergistically. At a numerical level, an order of magnitude change occurred by shifting from consensus (about 10000, Supplementary Tables 5.1) to model-driven significant detections, including DEGs and DE ncRNAs (about 1000, Supplementary Tables 8.1.1-5 and 8.2.1-8; Supplementary Fig. 8.1.1-2, 8.2.9-10 and 8.3.1). Data reduction also induced qualitative advantages with better biotype annotations (Supplementary Fig. 6.1.1.1-5, 8.4.1; Supplementary Tables 8.4.2-4), including pseudogenes (129) and related parental genes (145) (Supplementary Tables 5.3.1-2 and Supplementary Fig. 5.3.3-4).

**Figure 2:**
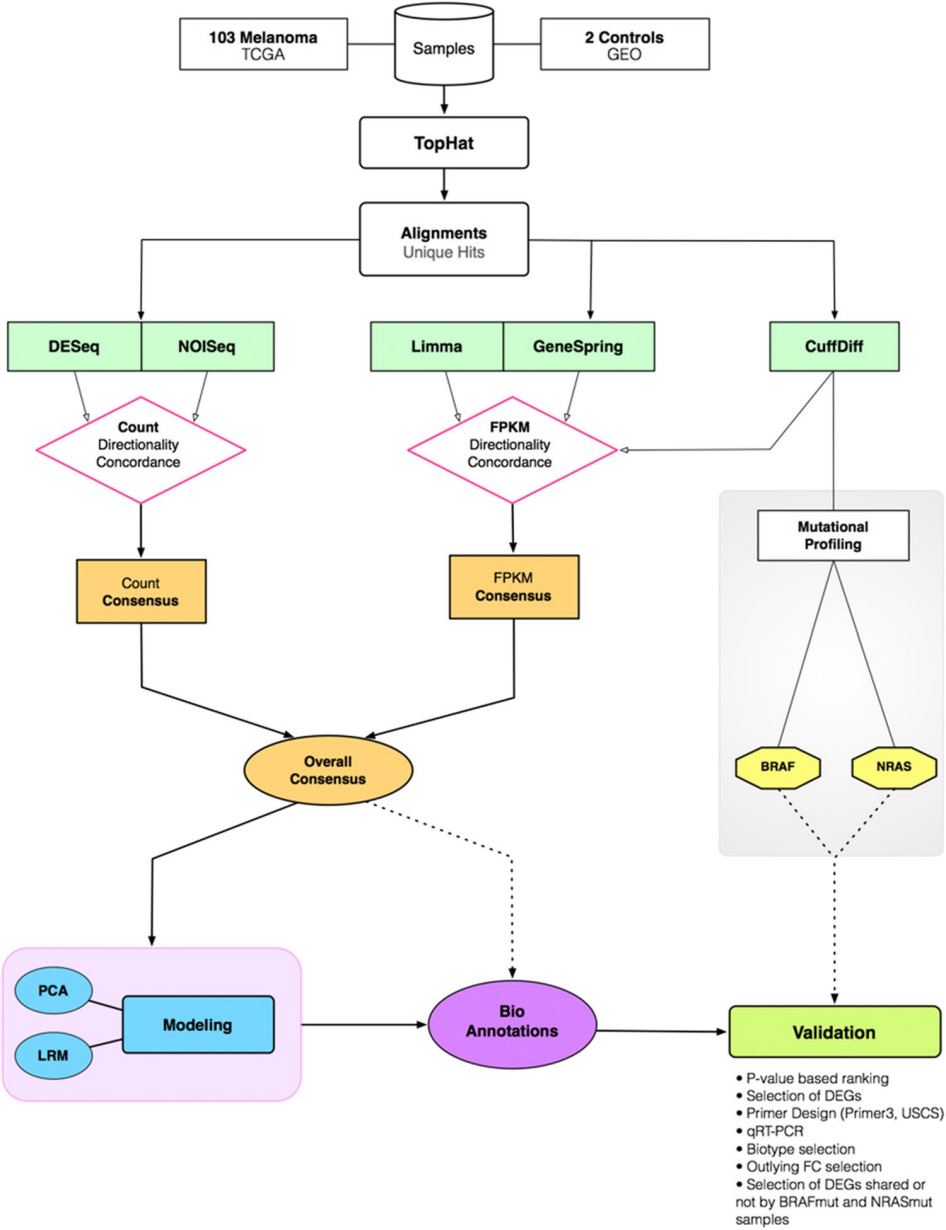
RNA-Seq flowchart. The samples were processed according to the five different methods that were selected, and whose quantifications included both read counts (simply the number of reads overlapping a given feature such as a gene) and fpkm (Fragments Per Kilobase of exon per Million reads). With the latter it is possible to compare genes of different lengths, and ‘per million reads’ means that a value normalized against the library size is obtained. The consensus occurred between significant detections in both read counts and fpkm scenarios, before reaching a global result (overall consensus). The mutational profile at the right side was implemented under simplified algorithmic conditions (CuffDiff), and for two major mutations (BRAF, NRAS) (Supplementary Tables 10.1-5). The validations refer to candidates coming from all the considered scenarios.

A summary of outcomes is presented in Fig. 2 with the flowchart of our RNA-Seq analyses: data generation, assemblies, mutational grouping and validations for subsets of outcomes from different data sources. Two standard quantifications were considered: read counts and FPKM (Fragments Per Kilobase Million) (Supplementary Tables S2_1-2). Other recent proposals^32^ aimed to yield transcript lists have not been used in this work, which is focused on gene-centric analysis. Thus, isoform detection and alternative splicing are not considered in this work.

### Differential Expression Profiling

The first set of significant detections, concerning both DEGs and DE-ncRNAs, were identified by the consensus between profiles using (see Fig. 1 and Fig. 2) DESeq^33^., NOISeq^34^., LIMMA^35^, CuffDiff^36^, and the licensed GeneSpring (V 13.0) (Supplementary Tables 3.1-5). These methods were selected to provide different types of quantifications, normalizations and statistical distributions.

**Figure 3:**
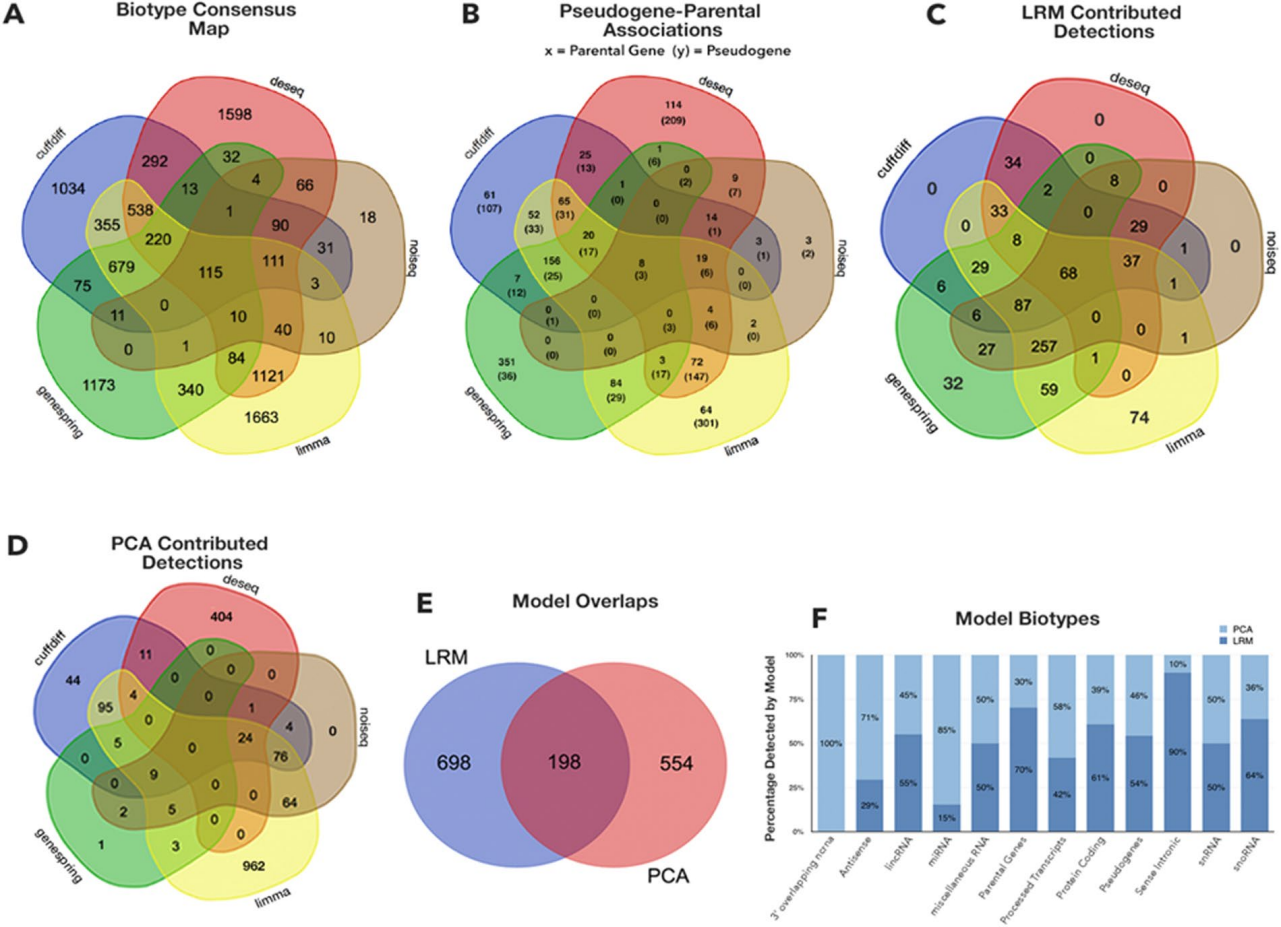
Venn diagrams: multiple detection scenarios. Detections refer to all DE biotypes. (**A**) Overall consensus. (**B**) Pseudogene-parental genes detections. (**C**) LRM-driven detections. (**D**) PCA-driven detections. (**E**) LRM vs PCA comparison. (**F**) Biotype classifications in each model. Thresholded coefficients are obtained by boxplot-derived criteria: from 1.5 x IQR for LRM (Supplementary Tables 8.2.5-8), and from most outlying values for PCA (Supplementary Fig. 8.1.1-2).

Due to small overlapping regions between the five methods (Fig. 3A), the inferable “DE-space” of significant DE biotype values appears questionable. The overlap degree from the employed methods might depend on the influences from non-controllable factors, for example samples, experiments, algorithms. Ambiguities appear, for instance with variable (high/low) log(FC) for identical calls obtained by different methods, indicating variation of signal intensity. This can be handled simply by averaging. When multiple methods detect with sign discordancy (overexpression vs underexpression), the uncertainty increases. Vice versa, detections identified by any particular method would need confirmation by other methods to be called significant.

The presence of well-separated DE-space regions representing the inferable DE-space, indicates sensitivity to the employed method. When assembling these outcomes, there is uncertainty about the confidence levels that may be assigned to such specific results. In principle, each method has localization power enabled by significance criteria (either conservative or liberal). These criteria depend on specific factors, such as distributional hypotheses, built-in normalization procedures, parametric settings, multiple corrections, etc., all exerting influence over the final detections. Generally speaking, local rather than global data features can bring good approximation power for inherently heterogeneous data, but likely poor generalization power. Note also that complex experimental data may lack robustness against possible under-sampling effects, which means that the spectrum of data features may be inadequately represented. Lastly, among the factors that exert negative impacts on inference, there are noise, algorithmic errors, and uncertain annotation of the bioentities (nomenclature from ENSEMBL as in Supplementary Tables 6.2.1-4) in addition to knowledge gaps and term redundancies typically present in GO and pathway tools. In general, a lack of substantial harmonization currently exists and leads to inconsistent results, even in presence of solid evidences. Of relevance the fact that our data-driven inference approach is both generalizable and scalable with dimensions and complexities, and tuned to increased accuracy in annotation tasks.

### Building the Ensemble from the Consensus

**Figure 4:**
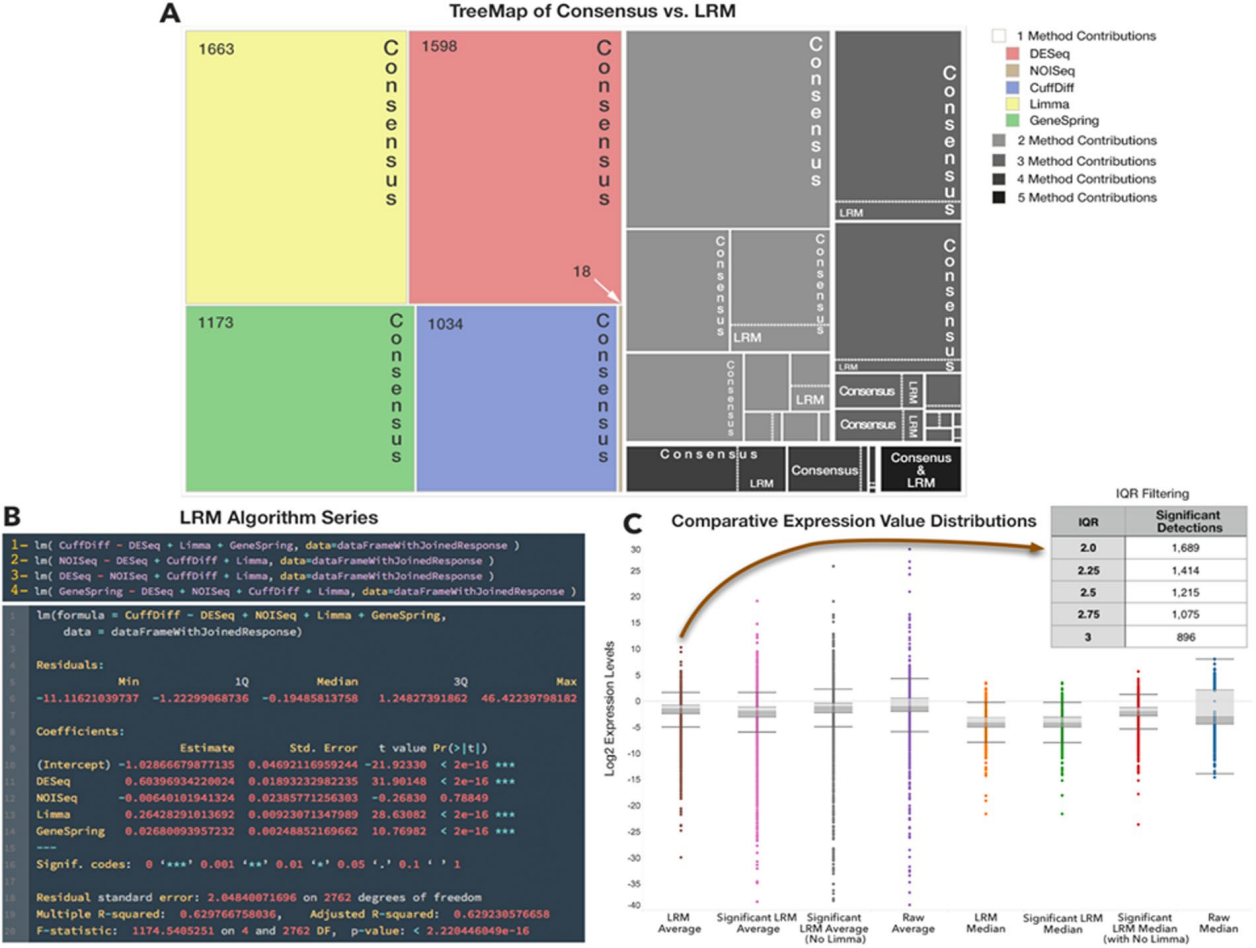
LRM output. Three panels are reported with reference to the performance of LRM: (**A**) shows a tree-map view of LRM vs consensus DE-space. The comparisons are localized in all regions in which both individual methods and combinations of methods (from 2 to 5) have performed and delivered their detections. LRM is visible always in just a small fraction of space. (**B**) presents the results screenshot of the LRM script in R. (**C**) shows comparisons between multiple LRM statistics used to establish the predicted profile, and given the adopted IQR levels (top right inset).

Figure 3 presents a sequence of Venn diagrams describing a map of detections, which indirectly tells how the methods saturate the DE-space with their derived profiles. First, a total of 9729 profiled bioentities is found from the overall consensus (Fig. 3A), with the innermost core sharing 115 bioentities. The core is surrounded by sets partially contributed by the five methods. Note that the most peripheral regions indicate the method-specific findings, which present the largest numbers, i.e. more than 1000 unique detections in each case (apart from NOISeq). Part of Fig. 3A detections, i.e. the ones relevant for pseudogenes and the associated protein coding genes classified as parental genes, are shown in Fig. 3B (see Supplementary Tables 8.5.3.1-2 and Figs 5.3.3–4 for the sequence analysis results testing the similarity between the two biotypes). Figure 3C shows another Venn diagram, identifying distinct and shared areas of detections (overall, 896 DE bioentities) that are specifically or jointly contributed by the RNA-Seq profiling methods, and cast within the LRM framework. The shared central core is halved compared to consensus (68 vs 115), and the peripheral regions with distinct detections are sparsified. Therefore, significantly estimated LRM coefficients select subsets of the detections, and reduce the impact of each method alone over the results. Figure 3D reports PCA detections (overall 752), then Fig. 3E compares LRM with PCA and identifies approximately one third of common detections. Figure 3F is for biotype cross-classifications. The observed limited overlap is relevant, in our view: substantial separation between the final selections from the model scenarios indicates that highly informative DE values are not necessarily brought by the component explaining much of the data variability, but rather depend on biotype associations that LRM establishes as significant. Figure 4 is centered on LRM, our chosen framework for ensemble modeling. The rationale is that regression offers an efficient setting for computation with large data sets, and an immediate interpretation of both the role of the parameters and the significance of the results (more in Discussion section).

Figure 4A describes a tree map view of the relative occupancy of the DE-space by LRM compared to consensus findings. By preserving proportions between LRM-selected regions and consensus ones, it clearly shows that LRM reduces enormously data redundancies, and likely also data complexities related to the algorithmic errors, noise effects, spurious detections etc. The ensemble strategy is clearly not panacea, as some of the lost DE-space may still be informative. However, the detected regions which appear shared between LRM and consensus are more consistently and robustly identified due to the possibility of merging synergistically method-specific profiles while quantitatively assessing the significance of contributions to calls obtained in model-driven way. Figure 4B presents for reproducibility purposes a screenshot of how LRM runs within R (more details are provided in Supplementary Tables 8.2.1-4). Here, a chosen profile is used as the response variable (Cuffdiff, in this case) and by the residual method-specific profiles (explanatory variables or regressors). All combinations are of course possible, and the algorithm indeed ensures their inclusion. Figure 4.C indicates the statistics used to evaluate the LRM performance, including both means and medians. Note that Limma turns out to generate the most outlying profile of the pool. This is relevant, as the ensemble modeling strategy primarily aims at reducing data redundancies and complexities, while improving the potential inferability and generalizability of the approach (even beyond the melanoma context).

Following the ensemble modeling strategy, it is important to understand how the LRM regions affect the system’s predictive performance. In particular, what method should be used for response and what for predictive purposes in the regression setting? To answer this question, we chose to run regression model multiple times according to the rotation of variables serving each role, response vs regressors. This scheme is known as Alternating Regression Model^37^ and measures how each variable contributes to the predicted response values according to a cross-validation scheme. Since each variable alternates its role at both sides of the equation, the final step involves computing statistics like mean and median values across both the replicated model runs and the predicted profiles. Intuitively, the LRM stability improves by rotating the variables, and we noted that an outlying role is played by Limma. We averaged the final profiles with and without it, but found the best results when Limma is excluded from the final averaging step. Its outlying values are not matched by other methods, meaning that no method contributes to its profile when Limma is used as the response. Instead, its predictive effects are retained (i.e. Limma as regressor) because the ensemble LRM models neutralizes them by assigning non-significance to the corresponding regression coefficients. Finally, gene and ncRNA signatures derived from each of the DE profiles required some control of variability, and we applied thresholding through the boxplots and the IQR measures applied to the estimated LRM coefficients. The stringency of the assigned thresholds affects the size of our final selections.

### Pseudogene-Parental Genes: relationships

**Figure 5:**
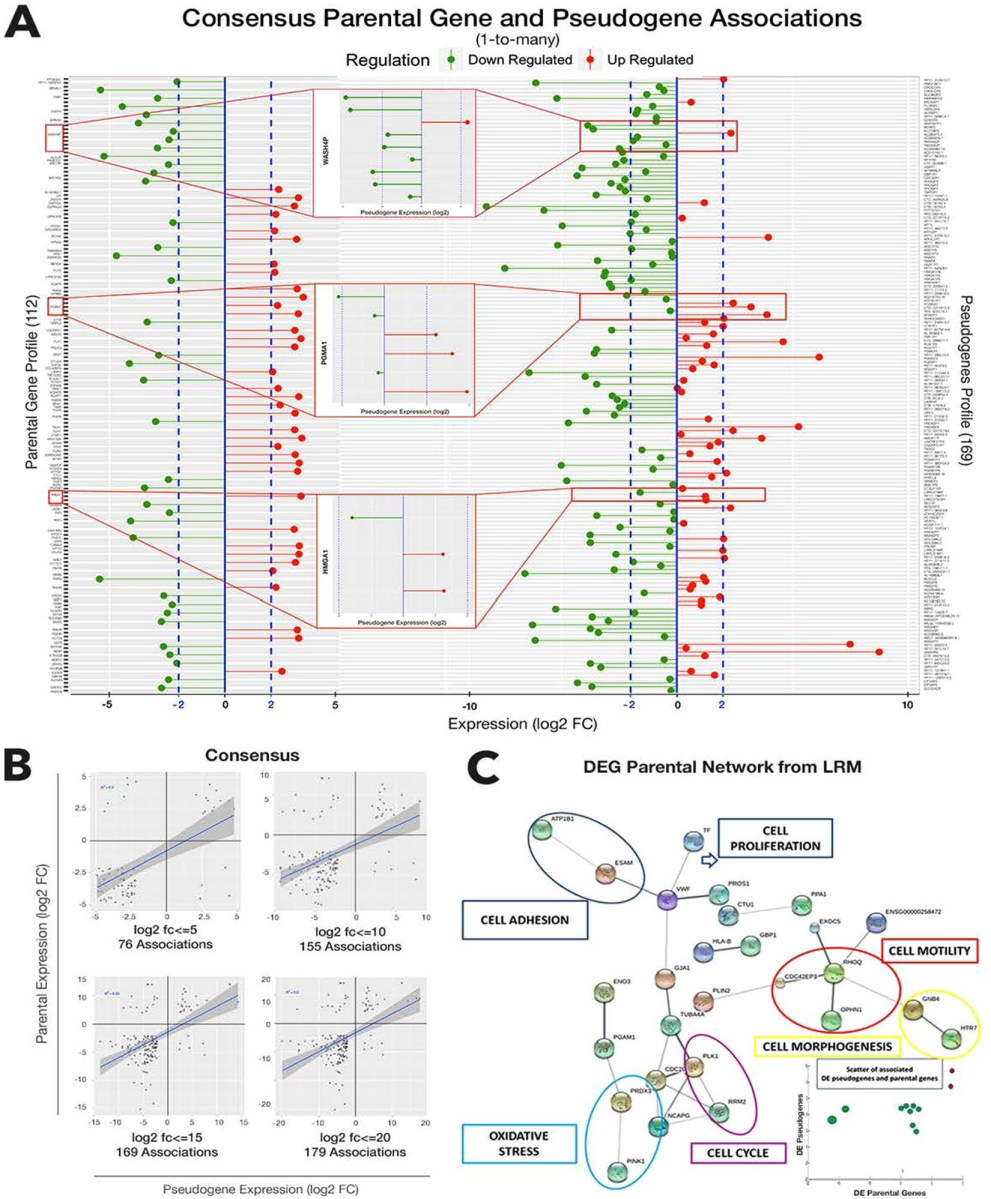
Pseudogene-parental gene analyses. (**A**) Joint profiles of pseudogenes and parental genes from consensus (with significance bars). Insets are biotype associations examples. (**B**) Scatters at various expression levels, and empirical correlation patterns. (**C**) Protein network of DE target parental genes with associated at least expressed pseudogenes (Supplementary Fig. 8.6.3 and Supplementary Table 8.6.5). Enlarged networks in Supplementary Fig. 8.6.4.

Figure 5 focuses on associations between DE pseudogenes and parental genes, whose patterns are determined by our computational pipeline. Such patterns have led to controversial conclusions in literature, due to correlative behavior between the two profiles that is inconsistently noticed. Figure 5A shows profiles measured within confidence bands for their expression levels. The central insets represent examples retrieved from a variety of target types. Figure 5B is about patterns from scatter plots measured at various log2(FC) expression levels. The slopes appear weak, but recurring across such levels. Despite a relatively low correlation, we note pattern persistency, which appears as a symptom of dynamics that are robust to random and outlying erratic effects. Figure 5C shows IQR-driven selections, depending on the statistics. We picked the simple average at the leftmost boxplot; each threshold choice brings a different number of significant detections (Supplementary Tables 8.2.5-8).

Interesting evidences appear from the network in Fig. 5C (similar methodology was recently proposed in a different disease context^38^). Formed from the DE parental genes associated with expressed pseudogenes (including DE), the network consists of interacting proteins retrieved from STRING V10.0^39^ (www.string-db.org), also annotated with GeneCards: the Human Gene Database^40^ (www.genecards.org). Given an intermediate level of confidence (0.4), from both experimental and database knowledge, these interactions occur between proteins relevant for cell proliferation, adhesion, motility, morphogenesis, together with cell cycle and oxidative stress.

The parental protein coding gene symbols determining the interactions are here reported with the symbol–indicating interaction: *ATP1B1-ESAM-VWF-TF-PROS1-GJA1-TUBA4A-CDC20-PLK1-RRM2-NCAPG; PINK1-PRDX3-PGAM1-ENO3; CTU1-PPA1*, *HLA-B-GBP1; PLIN2-CDC42EP3-RHOQ-EXOC5-OPHN1-GNB4-HTR7*. To some extent, these are all processes relevant to cancer, implying potential regulation underlying the target parental genes represented in the network and exerted by the either expressed or associated DE pseudogenes. When only DE pseudogenes are considered, we could observe a correlative scatter (Fig. 5C, bottom right inset). The few associations we found revealed concordance in both log(FC) size and sign (over/under expression). Note that some parental genes present multiple associated pseudogenes, but in general we found only one expressed example among multiple candidates (more details are provided in Supplementary Tables 8.6.1-5).

Interestingly, the PTEN- induced putative kinase 1 or PINK1, interacting with PRDX3, appears annotated with oxidative stress^41^, and is relevant because associated to both Parkinson disease and cancers^42,43^. In particular, melanoma is more frequently associated to Parkinson patients than other cancers^44^. PINK1 overexpression/underexpression respectively protects/sensitizes cells against/to stress. Also, PINK1 has a crucial role in mitochondrial homeostasis. Mitochondria are the major cellular sources of reactive oxygen species (ROS), being critically involved in many cellular processes, including energy production, metabolism, redox control, and programmed cell death. Their dysfunction is identifiable in numerous human diseases, including cancer^45,46^. Thus, PINK1 is essential for survival and a potential drug target due to its mediating role towards PTEN^47^. Despite the expression of PINK1 is up-regulated in melanoma with high metastatic potential, the molecular mechanisms by which PINK1 protects cells against apoptosis are unclear Notably, PINK1 is among the target parental genes that we have validated (see below).

### Bioannotations

#### Mutations

Among the mutations involved in melanoma pathogenesis, those repeatedly identified from BRAF and NRAS oncogenes have been investigated^48,49^. Two groups were obtained from both mutations, and are reported in Supplementary Tables 10. The mutated BRAF is V600E in 70–80% of all BRAF mutations in all cancers. Their relevance depends on the fact that targeted molecular therapies have been centered on BRAF, through BRAF inhibitors, and have included also combinations of oncogenic signaling cascade/pathway targets to improve long-term patient response and overall survival^50^. Mutations in NRAS, occurring at limited frequency compared to BRAF, were considered too. These two mutation types are almost always mutually exclusive. We accounted for the fact that approximately 50% of melanomas of all clinical types present an activating mutation in the BRAF oncogene, while about 20% of the cases show an activating mutation in NRAS, leading to the activation of the MAPK signaling pathway (cellular proliferation and survival depend on such pathway).

Previously, whole-genome sequencing of 25 metastatic melanomas and matched germline DNA revealed 11 genes significantly mutated, for BRAF in 16 samples and for NRAS in 9 samples^3^. The raw reads of SKCM samples were downloaded from TCGA site and were mapped on human genome using TopHat v2.0.11. SNPs were called by GATK 2.5-2-gf57256b. Non-synonymous mutations on V600 of BRAF were detected on 57 cases, but 19 were then discarded due to the low sequencing depth (<5x) on the mutation site, thus remaining with 38 cases. Non-synonymous mutations on Q61 of NRAS were detected on 17 cases, but 2 were then discarded due to the low sequencing depth (<5x) on the mutation site, thus remaining with 15 cases. Also, 33 cases have revealed no mutation on either V600 of BRAF or Q61 of NRAS, thus making the mutation-free group. The DEGs were detected by CuffDiff v2.2.1, with cutoff q-value < = 5% and log(FC) >1.2 between the BRAF-mutation group and the mutation-free group, and between the NRAS-mutation group and the mutation-free group. Overall, 1254 DEGs were detected in the former comparison, while 716 DEGs were detected in the latter one.

#### Pathways

We considered annotations from GO (Supplementary Fig. 6.1.2.1-5) and pathway terms (Table 1), and the lists generated by each individual profiling method (Supplementary Fig. 6.1.1.1-5) that formed the union of the consensus, together with the model-driven lists. ClueGo offers a selection of enriched terms. Note that each method-derived profile generated a list of terms enriched by gene sets whose biological significance is naturally affected by the DE space volume. Among the top-entries (sorted by enrichment) that were listed for each individual method (Table 1

**Table 1:** *Pathway terms* ranked by methods (left) and LRM. *is for cancer specific terms.

**DESeq**	**LRM**
*ECM-Receptor Interaction **Collagen Formation, Assembly of Collagen Fibrils, Collagen Biosynthesis Cytokine-Cytokine Receptor Interaction** *Cell Adhesion Molecules **(** ***CAMs*** **)** *PI3K-Akt Signaling Pathway **Protein Digestion and Absorption**	*Cancer Specific Terms (FDR-corrected P-value)
	Cell Adhesion Molecules (6.53E-10)ECM-Receptor Interaction (5.68E-06)Focal Adhesion (1.54E-05)PI3K-Akt Signaling Pathway (2.52E-03)p53 Signaling Pathway (9.31E-03)ECM Organization (3.41E-02)
**NOISeq**	**Top Scored Terms** (FDR-corrected P-value)
* ***ECM-Receptor Interaction*** **Collagen formation, Assembly of Collagen Fibrils, Collagen Biosynthesis, Protein Digestion and Absorption** *Cell adhesion molecules **(** ***CAMs*** **)Leishmaniasis, Interferon Gamma Signaling**	Phagosome (7.42E-12)Complement System (8.09E-11)Protein Digestion and Absorption (6.53E-10)Leishmaniasis (3.59-08)Collagen Formation (1.26E-08)Allograft Rejection (1.24E-07)Viral myocarditis (1.55E-07)Graft-versus-host disease (1.75E-07)Type I diabetes mellitus (3.19-07)Asthma (1.45E-07)Rheumatoid Arthritis (6.46E-06)Cell Cycle (5.68E-06)Autoimmune thyroid disease (4.02E-05)IgA Intestinal Immune Network Production (1.31E-05)Tuberculosis (4.31E-05)Hematopoietic cell lineage (2.02E-04)Pertussis (2.12E-04)Systemic Lupus Erythematosus (2.60E-04)HTLV-I infection (2.88E-03)Spinal Cord Injury (1.19E-03)Inflammatory Bowel Disease (IBD) (2.16E-03)Toxoplasmosis (5.02E-03)Natural killer cell mediated cytotoxicity (6.95E-03)Insulin Processing (2.43E-03)Leukocyte transendothelial migration (1.27E-02)
**CuffDiff**	
*ECM-Receptor Interaction **Immunoregulatory Interactions between a Lymphoid and a non-Lymphoid cell** *Cell adhesion molecules **(** ***CAMs*** **)Collagen formation, Hemostasis Assembly of Collagen Fibrils Natural killer Cell Mediated Cytotoxicity** *Rap1 signaling pathway	
**Limma**	
*ECM-Receptor Interaction **Hemostasis, Complement and Coagulation Cascades, Collagen formation, Cyclin B2 Mediated Events, Cytokine-Cytokine Receptor Interaction, Collagen Biosynthesis**	
**GeneSpring**	
**Cell Cycle, Mitotic Prometaphase, Mitotic Metaphase and Anaphase, Separation of Sister Chromatids, Mitotic Anaphase, Resolution of Sister Chromatid Cohesion**	
**PCA**	
**Rheumatoid Arthritis, Phosphorylation CD3 and TCR, Hematopoietic Cell Lineage, TCR Signaling, ZAP-70 Translocation, Cytokine-cytokine receptor interaction** *PD-1 signaling	

, left), we found some interesting terms (note that colored entries identify cancer-relevant terms). The ECM Receptor Interaction appears in four over six lists (including PCA) followed by the CAM (cell adhesion) term in three over six lists. PI3K-Akt, Rap1, PD-1 appear in three separate lists.

By looking at the LRM terms (Table 1, right), note that a change of significance can occur in both terms and gene sets enriching for them, due to different criteria of selection. Some terms are known to be strong players in melanoma, and we reported then at the top. CAM, ECM and PI3K-Akt appear specifically as before, together with Focal Adhesion and p53. The top-score list includes a variety of terms. More in detail, PD-1 is an inhibitory receptor found in T-cells, B-cells and myeloid cells, highly expressed under certain conditions in which important immune system modulators (T/B cells etc.) are activated. Of interest the involvement of kinases that are inhibited by PD-1 after binding to ligands, and thus preventing overstimulation of the immune response^51–53^. Its ligands have different expression patterns, and interactions with them attenuate the immune response and protect tumor cells from cytotoxic T-cells. PD-1 is also expressed in patients with metastatic melanoma particularly within the tumor microenvironment, suggesting that the immune response to melanoma is inhibited. PD-1 (likewise PD-L1) is therefore currently considered a primary therapeutic target. The PI3K (phosphinositide 3-kinase)–Akt pathway is studied to decipher molecular pathogenesis, heterogeneity and resistance mechanisms, offering emerging leading targets for compounds^54,55^_._ Melanomagenesis involves ECM signaling, p53, RAS/RAF/MEK/ERK, among others. It is also interesting the presence of the Complement system (LRM, but also Limma), an essential element of the innate immune response that reacts as a first line of defense against pathogens and in response to molecular patterns associated with abnormalities in host cells and extracellular environment^56^.

#### Biotypes & Gene families

We expanded the annotations to include gene families and biotype decompositions (and more details in Supplementary Tables 7.1 and 8.5.1). Here we include further characteristics. In biotypes (Table 2) we list ENSEMBL classes, and we may note the prevalence of antisense, lincRNA and pseudogenes over the other classes, with exclusion of protein coding genes (parental genes are a subset of them, reported apart to emphasize their target role). Together these three classes of ncRNAs cover in some cases about one fourth (DESeq, LRM) or even one third (Limma) of the total number of listed DE bioentities. Gene families (Table 3) instead enriched qualitatively the descriptions of many of the protein coding genes that were classified in the previous lists. It is interesting to verify the sensitivity of such classifications by the employed methods, observing that DESeq and Limma rank first in four classes each, and again Limma and CuffDiff rank second in four and three classes, respectively. The two models, PCA and LRM, are of course subject to more restrictions compared to the consensus, and delivered overall just a fraction of entries in each class here considered (the only exception being NOISeq, which appears very conservative).

**Table 2:** Classification by Model Biotypes.

Biotypes	DESeq	NOISeq	CuffDiff	Limma	GeneSpring	LRM	PCA
3′ Overlapping ncRNA	4	0	1	5	1	0	1
Antisense	412	14	197	646	150	65	157
lincRNA	349	17	243	574	96	50	41
miRNA	14	0	25	5	11	2	11
misc_RNA	6	0	51	12	16	1	1
Parental Genes	355	62	431	549	631	115	49
Processed Transcript	213	8	169	302	55	26	36
Protein Coding	2,811	438	2,390	2,962	2,248	648	418
Pseudogene	468	32	250	618	151	85	72
Sense Intronic	25	0	9	107	14	9	1
Sense Overlapping	16	0	5	26	2	0	0
snRNA	2	0	2	4	2	2	2
snoRNA	6	0	1	23	12	7	4
**Total**	**4,681**	**571**	**3,774**	**5,833**	**3,389**	**896**	**752**

**Table 3:** Gene Families Associated with Protein Coding Genes.

Gene Family	DESeq	NOISeq	CuffDiff	Limma	GeneSpring	PCA	LRM
Tumor Suppressors	4	0	8	9	6	1	1
Oncogenes	52	5	57	65	56	12	14
Translocated Cancer Genes	48	5	54	59	51	10	14
Protein Kinases	70	7	79	101	82	15	18
Cell Differentiation Markers	112	27	94	105	45	23	22
Homeodomain Proteins	48	2	18	36	7	4	3
Transcription Factors	179	11	127	176	163	23	16
Cytokines and Growth Factors	127	25	83	83	26	23	13
**Total**	**640**	**82**	**520**	**634**	**436**	**111**	**101**

Notes: number of detections in each cell refers to differentially expressed values. ENSEMBL classification (Table 2); other annotations (Table 3).

#### Validations

**Figure 6:**
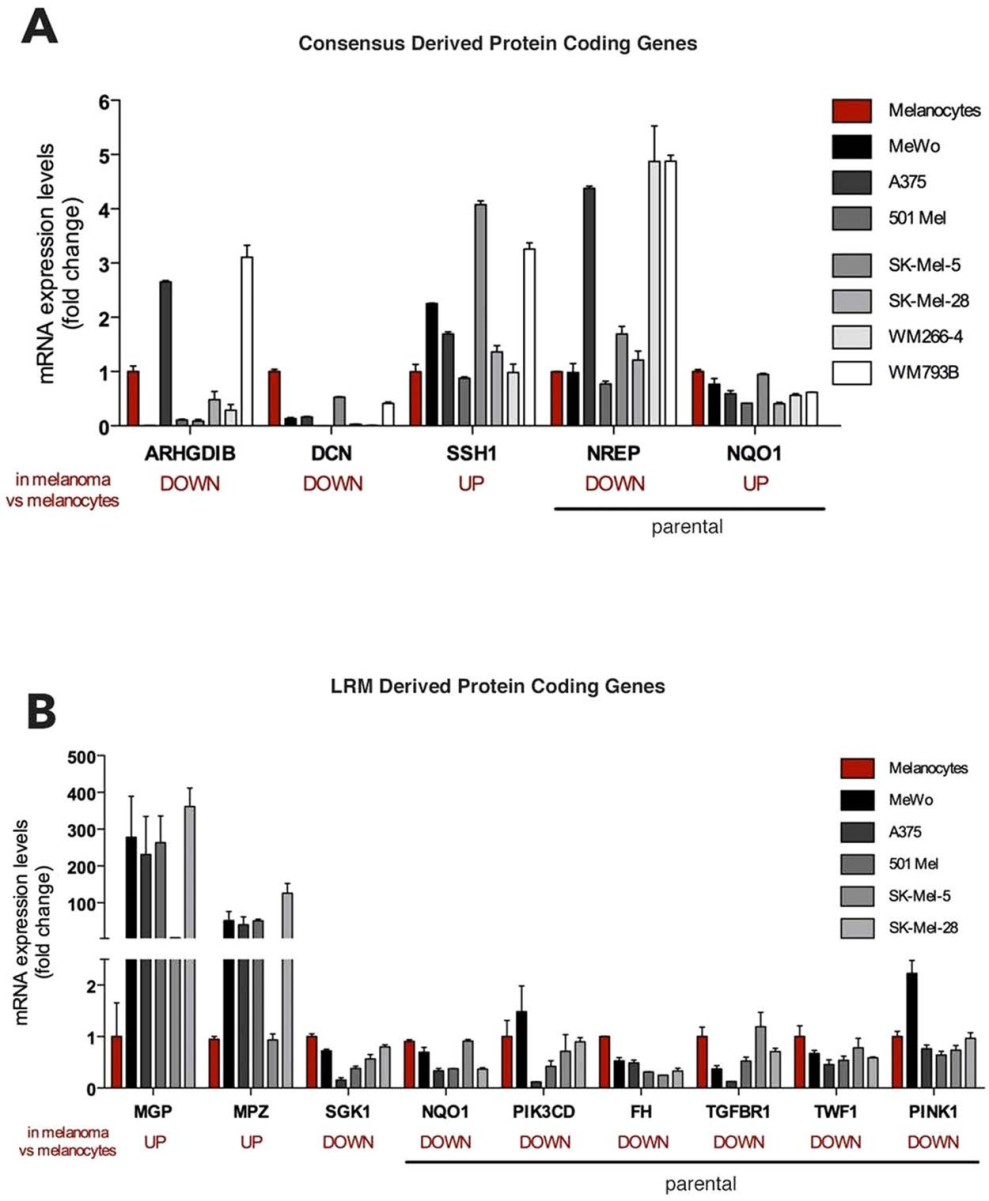
Validated evidences: from consensus (**A**) and LRM (**B**) **(**details in Supplementary Fig. 9.1 and Supplementary Text 9.2).

We first considered consensus data (Fig. 6A), and made a few selections. Then, we considered LRM-derived selections (Fig. 6B). Candidate selections were organized in a list including measurements and bioannotations for protein coding genes (details in Supplementary Fig. 9.1 and Supplementary Text 9.2). Focus went on the list of DE pseudogenes and DE parental genes, together with other DEGs. As consensus involved the application of five different RNA-Seq methods, the selection considered up- or down-regulated DEGs and delivered three pseudogenes and 111 DEGs, including eight parental ones. All genes were ranked on the basis of the corrected p-value attributed by each individual algorithm. As an example, we ranked 115 DEGs on the basis of the corrected p-value attributed by Exonic DESeq, and then assigned score 1 to the lowest one, 2 to the second lowest one, and so on. This procedure was repeated using the corrected p-values attributed by the other four algorithms. Finally, we summed up all scores together and calculated a final score. The list was scanned top-down in search for candidates for which the design of specific primers was possible. The chosen candidates were at the end three DEGs: *ARHGDIB*, *DCN*, *SSH1*, and two parental genes: *NQO1* and *NREP*. These genes were DE in melanoma samples compared to melanocytes. Therefore, in order to confirm these results, we measured their expression levels in melanocytes and melanoma cell lines using rtPCR. Both the down-regulation of *ARHGDIB* and *DCN*, and the up-regulation of *SSH1* were confirmed. However, we did not confirm the up- and down-regulation of *NQO1* and *NREP*, respectively. We then considered the LRM outcomes. For the selection of the genes to be validated by rtPCR, we prioritized parental genes as the reference biotype, then we selected genes that encode for proteins belonging to cancer-related pathways such as the PI3K-AKT pathway (SGK1 and PIK3CD) or the cell adhesion pathway (MPZ), for kinases (e.g. PINK1, TGFBR1 and TWF1) or for tumor suppressors (FH). The chosen candidates are nine protein coding genes, six of which are parental genes. Using rtPCR we confirmed down-regulation for *SGK1*, *NQO1*, *PIK3CD*, *FH*, *TGFBR1*, *TWF1*, *PINK1* and up-regulation for *MGP* and *MPZ*. Note in particular the case of *NQO1*, initially not validated according to the consensus, but then validated according to LRM, this of course depending on the different selection operated in the two cases.

## Discussion

Knowledge advances in cancer genome and transcriptome studies depend, among other factors, on the integration between experimental and computational resources and skills. Such type of integration operates through data-customized bioinformatics pipelines. Despite a substantial variety of proposed methods, careful literature review suggests that a restricted number of them are routinely involved in major applications, thus making them standard approaches. Such process generates a sort of biased automatism through which the confidence assigned to results impacts cancer studies, but is not supported by comparative evaluations between methods or tests measuring sensitivity of results. Literature has devoted attention to different normalization techniques, for instance, without demonstrating solid criteria for preference between methods. The field keeps evolving, naturally enough, but with uncertainty about our detection accuracy. Additionally, and in cancer studies especially, some of the existing tools may be too conservative, and new supersets of data could be needed to cross-validate analyses and findings. This consideration is applicable to other studies^57^. Our contribution turns to the search for possible ways of selecting evidence that may yield a solid knowledge base specifically for melanoma, but not limited to this cancer.

Additionally, we focus on protein coding genes and ncRNAs, and in particular pseudogene-parental gene associations that were retrieved from the SKCM data. The relevance is clear: despite pseudogenes are only dysfunctional copies of protein-coding genes, pseudogene transcription has a pervasive presence in the human genome^58^. The correlation patterns between intronic pseudogene expressions and unrelated host gene expression values were not revealed, and no correlative patterns with their parental genes appeared from the 10 melanoma samples that were included in the study^58^. Correlation between pseudogenes and parental genes was instead found for about 65% of pairs to be positive, depending also on the number of predicted miRNA targeting sites^23^. Considering another scale, it has been demonstrated a combined regulative action of pseudogenes over a miRNA target, the tumor suppressor gene PTEN expression, through the mechanism known as ceRNA (competing endogenous RNA)^59,60^. Overall, from both biological and computational viewpoints, the sequence similarity between pseudogenes and parental genes contributes to the complexity of both sequencing and gene expression profiling, and even validation. This global complexity calls for additional research^61^. We have indicated association patterns showing a certain consistency. However, limitations are naturally present and they mainly concern the fact that detecting pseudogene transcripts is a complicated matter with a few critical factors to keep in mind:

- Technological limitations: they affect the real detection power and may act as confounders for predictive algorithms, especially when the similarity of pseudogenes with parental genes is high;
- Computational problems with regard to alignment, i.e. reads that align to multiple genomic locations. *RNA-Seq* presents read mapping uncertainty causing loss of both information and coverage;
- Assembly limitations: a limited resolution and the fact that transcript variants from the same gene may have exons in common, can cause ambiguity difficult to resolve;
- Sequencing depth: it is well-known that low or broadly expressed genes are not strongly supported by *RNA-Seq* as only abundant transcripts are fully assembled;
- Other problems come from sequencing non-uniformity, presence of novel isoforms (alternatively spliced transcripts), quantification of expression levels, and harmonization of data normalized according to various criteria, for instance form experiments with different sequencing depth.

Following the ideal path of transforming observations (data) into model outcomes (predictions) to reconcile system’s dynamics, a limitation is that moving from observations to inference, and then to validation, is not usually a linear path. In our view, the strategy of moving from a low- to a high-confidence set of DE bioentities develops along two routes: a) Empirical approximation through PCA, i.e. projecting the data to perform de-noising and extracting the prevailing signal in terms of variability; b) the path of LRM, which exploits the prediction power of each method according to its significant contribution to a response profile. Therefore, we may aim at approximating with acceptable accuracy the recoverable system’s dynamics^62^. Ensemble modeling improves results by targeting a multitude of significant evidences, which here are the detections obtained from RNA-Seq data, and it achieves superior accuracy than any individual method. Benefits have been shown here in terms of bioannotations (GO categories, pathways, gene families), and validations.

Naturally enough, LRM encounters known limiting factors, such as the possible violation of linearity, an excess of variability across the covariates, and the risk of overfitting. Methodological solutions for such problems that go beyond naïve linear model approaches are well-known (ridge regression, regularized solutions, LASSO, LARS, etc^63^.). However, we have proven that the stability of the system can be promptly checked, and corrections can be made to improve the overall performance. Our regression framework simply stacks together all detections in a matrix with rows assigned to the distinct measurements (outcomes) and columns hosting the ‘explanatory variables’ (predictors). The contribution of each method is therefore mediated by the linear combination which determines the optimal prediction for each DE entry listed as the ‘dependent variable”. This way, an ensemble predictor built-in in a regression framework can be provided by averaging effects across the rows such that all model variables are re-modulated by significant coefficient estimates aimed at tuning the prediction power.

Cancer systems present uncertainties in model structures and parameter values. One would like to measure not only the goodness of fit, but also the complexity of a model. Reducing the complexity would help identifying core dynamics, thus increasing the chances of best fit.

The analysis of ensembles of models which vary in parameter values is a powerful strategy for reducing ambiguity in systems biology and medicine. The model ensemble may encompass different structures to capture the systems dynamics. Whether these dynamics are stylized or simplified is relevant, as cancers are very partially known complex systems. Therefore, models may be sloppy^64^. Sloppiness is not simply concerning a lack of data such that trivial parametrizations lead to non-identifiability of the parameters (undetermined systems), but might involve more subtle aspects, such as redundancy or compensatory effects between sets of parameters^65^. It is also likely that both important and not important parameters may be often hard to separate.

To conclude, we recall a very useful methodological principle that we have pursued through the suggested ensemble approach: along the path of ‘weakening inference algorithms’^66^, and in order to solve complex problems such as dimensionality reduction and identification of coresets, being these gene sets for example, an effective strategy requires the concentration of the total information into salient data features. Although sub-optimal (loss of DEGs and DE-ncRNAs, residual multicollinearity, spurious correlations from confounding effects etc.), this idea works under simple model assumptions, such as those here enabled by regressions. The great value of the performed analyses is to increase the ‘data strength’ and through this step obtain a high-confidence compendium of selected bio-entities.

## Methods

### RNA-Seq Samples

103 primary tumor Cutaneous Melanoma (SKCM) samples were downloaded from the Cancer Genome Atlas (TCGA) CGHub repository (https://tcga-data.nci.nih.gov/tcga ). 2 control samples were also downloaded from the Gene Expression Omnibus (GEO) repository^67^, from a study of with data accessible at NCBI GEO database with accession GSE37169^68^. The total number of 105 samples were downloaded in FASTQ format^69^.

### Sample Processing

All sample reads were mapped to the Ensembl^70^ human genome reference (Genome Reference Consortium build 37, Ensembl v.72) using the TopHat 2 spliced aligner^71^. The human genome reference sequence was prepared for mapping by collating and indexing the 25 standard chromosomes (22 autosomes, X and Y Chromosome, and the Mitochondrial chromosome) from Ensembl. The ‘bowtie2-build’ indexer program from Bowtie2^72^ was used as the indexing step and the TopHat2 spliced mapper was used to independently map each of the sample reads from TCGA and GEO. Tophat2 requested a GTF annotation file (http://mblab.wustl.edu/GTF22.html) from Ensembl. After mapping, Samtools^73^ was used to process the output files from the 105 samples, thus converted to a format (.BAM) suitable for downstream quality assurance and analysis steps.

### Quality Control

Each sample was inspected to detect possible anomalies. Issues emerged with the percentage of mapped reads in two of the TCGA tumor samples, therefore excluded. The residual 101 TCGA tumor samples, and the two controls (from GEO), were thus analyzed.

### Mapping Qualities

Mapped reads for each sample were sorted and binned based on their mapping quality (MAPQ) values. SamTools was used to filter reads based on MAPQ values. TopHat2 bins read alignments based on 5 MAPQ classes: values of 0, 1, 2, and 3 are assigned for read alignments with multiple hits of 10, 4-9, 3, and 2 locations, respectively; a value of 255 is for unique alignments. Only unique read alignments for each sample were extracted from the output files, and used for downstream analysis.

### Expression Quantification

Unique read mappings from the 103 samples were used to create expression units for each gene in the Ensembl v.72 database, this for each sample. Each gene in Ensembl was quantified using FPKM and Read Count units^74^. We applied two constraints: genes showing an FPKM greater than 1 and showing >10 reads in at least 10% of samples were included for downstream analysis, in both conditions. Different combinations and permutations of FPKM and number of reads at variable percentages were tested (details in Supplementary Table 2.1-2 and Supplementary Fig. 4.1-2).

- **FPKM**. FPKM units were calculated using CuffDiff. The CummeRbund^75^ R package to process and extract the FPKM data. A table of FPKM values was created for downstream analysis, containing genes as rows and samples as columns.
- **Read Counts**. Read count units were calculated using the HTSeq software^76^ for both exonic reads (reads mapping to exons only) and intronic reads (reads mapping to both exons and introns). A table of read count values was created for downstream analysis, containing genes as rows and samples as columns (Supplementary Tables 5.4.1-2 report intron results too).

### Pseudogene-Parental Genes

The criteria for assessing transcribed evidence are mainly two: 1) Reads mapped to the pseudogene sequence and not to the parental gene; 2) Reads mapped to both the pseudogene and to the parental gene, but with lower sequence similarity (<90%). Target parental genes were identified by aligning the pseudogene sequences using BLAST against a database of the protein coding CDNA sequences from Ensembl (v. 72). The best hit matches for a pseudogene sequence were selected based on e-value scores, and the best overall hit for a pseudogene was selected as its parental gene. BLAST results with e-value scores can be found in Supplementary Table 1.1. In total, 13,151 candidate associations were found from the BLAST results. After removing duplicates, 4,035 unique protein coding parental genes were identified (Supplementary Table 1.2). Parental gene and child pseudogene associations can be found from consensus (Supplementary Tables 8.5.3.1-2 and Supplementary Fig. 5.3.3-4).

### Differential expression

FPKM and read count values were used for testing relative differential expression between the TCGA primary tumor samples and the GEO controls. Five (5) Methods were employed with different (FPKM or read counts) units as input. FPKM-based chosen methods were: limma from Bioconductor^77^, GeneSpring (http://www.genomics.agilent.com/), and CuffDiff. The chosen read count methods were DESeq and NOISeq (both in Bioconductor). Each method was run using the standard protocol and parameters available in their documentation, and output p-values were corrected for multiple comparisons using false discovery rate (FDR).

### Methods for Profiling

Each of the differential expression methods pooled the two conditions tumor and control together (Supplementary Tables 5.1.1-5).

- **DESeq**. The R package (v. 1.18.0) performs analysis of read counts from HTSeq by estimating variance-mean dependencies, normalizing by an estimate of the effective library size, and testing for differential expression using a negative binomial distribution.
- **NOISeq**. The NOISeq package (v. 2.8.0) is a non-parametric method for analyzing read count data from HTSeq. There are no assumptions about data distributions, and normalization of count data is by using a trimmed mean of M-values (TMM)^60^.
- **CuffDiff**. The CuffDiff software (v. 2.2.1) models the variability of a gene’s RNA-Seq measurements by considering RNA-Seq fragment mappings and the gene’s splicing structure. CuffDiff normalizes the library size by using a quartile normalization, and estimates the replicate dispersion with a Poisson model.
- **GeneSpring**. The GeneSpring software (v. 13.0) (Agilent Technologies) analyzed FPKM values obtained from CummeRbund. A standard workflow for expression data was used, and no further normalization was applied. Tests for differential expression were calculated using a Moderated T-Test.
- **limma**. The limma package (v. 3.22.6) analyzed the FPKM data from CummeRbund. The “classic” workflow was performed, i.e., no ‘voom’ transformation was applied. Tests for differential expression were calculated using a Moderated T-Test.

### Pathway Analysis

DEGs from each algorithm were analyzed for pathway enrichment using the ClueGO^78^ plug-in for Cytoscape^79^ (http://apps.cytoscape.org/apps/cluego) The ClueGO db for Gene Ontology (GO)^80^, Kyoto Encyclopedia of Genes and Genomes (KEGG)^81^, WikiPathways^82^, and Reactome^83^ were used for pathway enrichment analysis with default values, with the GO analysis being run independently from the other databases. Several molecule pathways are associated with melanoma, and some with clinical relevance (see a comprehensive list in^84^. Statistical analysis included enrichment performed with Benjamini-Hochberg p-value correction. Details on various enrichments are reported in Supplementary Tables 8.4.2-4.

### Principal Component Analysis (PCA)

The function ‘prcomp()’ from the R “stats” package was used to perform PCA on both the counts and the FPKMs, thus covering profiles from all the methods. PCA coefficients from the first PC were used to perform box-plot thresholding (data points outside the maximum and minimum whisker boundaries were kept as outliers).

### Linear Regression Model (LRM)

A merged matrix of log2(FC) from the differential expression results of all methods was created and used as input to LRM. The matrix consisted of genes (rows) and algorithms (columns), with each cell containing gene or ncRNA values. Values that were not identified by a method were flagged as missing in the matrix. The standard form “y ~ model” is applied, by the R function “lm()” designed to fit the LRM according to:

$${\rm{CuffDiff}} \sim {\rm{DESeq}}+{\rm{NOISeq}}+{\rm{Limma}}+{\rm{GeneSpring}}$$

The above simply denotes that the outcome is assigned by one method, say CuffDiff, establishing the model response variable associated to explanatory variables forming linear combination of values obtained with DESeq, NOISeq, Limma, and GeneSpring (i.e. the other methods). The placement of such methods as response vs explanatory variable is subject to rotation, according to the idea of Alternating Regression Modeling. Predictions by the model were obtained using the R function “predict.lm()”, then filtering by a suitable multiplier of the interquartile range (IQR). Preference went to predicted values outside 3*IQR range.

### Further notes on models

#### PCA-driven selections

In principle, PCA primarily finds the data directions explaining most of its variation. A dataset D, normalized into D’, has covariance matrix computed as COV(D’), and its eigenvectors (sorted from the largest to the smallest eigenvalues) represent the PCs. The principal component coefficients, also known as loadings, are to be found. Given, say, the *n*-by-*p* data matrix D, rows of D correspond to observations and columns correspond to variables. The coefficient matrix is *p*-by-*p*. Each column contains coefficients for one PC, columns in descending order of component variance. Equivalently, the PCs return information contained in the projected data through the correspondingly estimated coefficients, with the highest relevance assigned to the direction of maximal variance (or principal direction). In our setting, the tumor baseline signal is contrasted with the control data, and the noise is reduced by shrinkage operated from the boxplots. Therefore, each method is subjected to extraction of the first component (PC1), and a set of boxplot outliers is obtained in each case.

#### LRM-driven selections

With regression, thresholding is applied via the interquartile range or IQR, a measure derived from boxplots. Optimality would imply some assumptions, a main one being predictors’ independence. This assumption does not usually hold, and sub-optimal conditions apply. As our setting presents intersections among the identifications obtained from five methods (e.g. overlapping sets), the emerging gene or ncRNA signatures must be confronted with the inevitable collinearity between regressors, i.e. the method-dependent profiles. Such redundancy may be reduced by enforcing shrinkage to the predicted values, through a robust selection rule, i.e. IQR × 1.5 (2 or 3). The selections represent linear combinations of multiple profiles.

#### Model selection

There might be a subset of predictors relatively more important than the rest. One strategy is to insert penalties in the model, useful to control the number of predictors involved. As an alternative, we proposed an ARM model type, one working by rotating the dependent and independent variables. Once an initial response is identified with one of the variables, the residual variables play the role of regressors/predictors for the response. It is a sort of cross- or hold-out validation principle applied to assess the goodness of fit of each method. Repeating the regression generates vectors of fitted values that need to be averaged at the end, thus yielding the final consensus DE vector. Note two aspects: (a) The dimension of each response varies depending on the profile to predict. The matrix of regressors will adapt to the response’s dimension by only matching selected response values; (b) Estimates of the regression coefficients guide the selection of variables (i.e. if the coefficients are significant, then the variables will be considered to contribute to the fitted values, otherwise they will be excluded).

Note that LIMMA inclusion revealed not significant regressors. Thus, LIMMA fitted values do not benefit from regression modeling, under the given hypotheses. This means that by delivering a much wider but different profile than other methods, LIMMA covers the gene space in regions unexplored by the other methods. However, this subspace is uncertain in terms of number of false positives. We cautiously keep LIMMA out of the final average for the fitted values. The LIMMA anomaly was also emphasized with the use of PCA, and in that case too the bias introduced was potentially too high to keep LIMMA’s evidences stacked with the other ones. In terms of final consensus DE profile, we obtained comparative results with crude average of outcome DEGs, total averages from regression, and fitted averages obtained from ARM with and without LIMMA. The most balanced distribution is always obtained in the last scenario.

#### Data Availability & Software

All software and datasets can be obtained at the Center for Computational Science’s GitHub repository, available at the following URL: https://github.com/ccs-bio/melanoma-transcriptomics. At this repository, expression tables (FPKM and read count values) and scripts (Python, Bash, R, etc.) for both RNA-Seq sample alignment and filtering to extract unique read mappings are reported. R scripts for differential expression can also be found for DESeq, NOISeq, and limma. A bash shell script is also included with the CuffDiff command call, along with the R script for processing the data with CummeRbund. The experimental grouping for GeneSpring is also available., as well as scripts for visualizations. All source code will be released under the GPL v.3 license.

#### Ensembl Annotations

The analysis was based on the Ensembl annotations version 72. Ensembl releases versions each year (Suppl Fig. S6_2_4), and the current version is 86. Version 72 (Supplementary Table 6.2.1) of the annotations contained a total of 62,893 genes, while version 86 (Supplementary Table 6.2.2) contains 58,051. Comparing both sets of annotations we found that 51,858 (82.45%) genes are contained in both, while 11,035 (17.55%) of the version 72 genes are not present in the current version, v.86. A comparison between genes present in the latest version of the annotations relative to version 72 is included in Supplementary Table 6.2.3)

### Validations

#### Cell lines

Cells were grown at 37 °C in a humidified atmosphere with 5% CO2. All the media used for cell culturing are reported in^85^, and were supplemented with 1% Penicillin/Streptomycin (Euroclone; Milano, Italy). The mutational status of BRAF and NRAS is reported in^85^ too.

#### Sequence retrieval & alignment

The cDNA sequences of the genes of interest were obtained from Ensemble, inserting the code available in the tables. In order to align sequences and detect their homology CLC Sequence Viewer 7 was used.

#### Primers

Primer design was performed using Primer3 (http://primer3.ut.ee/), while UCSC in silico PCR (https://genome.ucsc.edu/cgi-bin/hgPc)rwas used to confirm the specificity of primers (absence of off-targets). All primers were purchased from Primm srl (Milano, Italy). Sequences are reported in Table 1-4 and Supplementary Text 9.3.

#### RNA extraction and retrotranscription

RNA was extracted using QIAzol (Qiagen; Venlo, Netherlands), following the manufacturer’s instructions. 1 μg of total RNA was treated with DNAse I, amplification grade (Invitrogen; Carlsbad, California) following the manufacturer’s protocol. 0.5 μg of RNA treated with DNAse I was retrotranscribed on a S1000 Thermal Cycler (Bio-Rad; Hercules, California) using iScript cDNA Synthesis Kit (Bio-Rad; Hercules, California). The successful retrotranscription and the absence of contaminating genomic DNA were routinely checked through a control PCR in which exon-spanning primers for ATPA1 gene are used. cDNA was diluted (1:4) and used for qRT-PCR.

#### qRT-PCR

1 μL of cDNA and appropriate primers (Table 1) were used for qRT-PCR reactions, using SsoAdvanced Universal Supermix (Bio-Rad; Hercules, California) on a CFX96 Real-Time System (Bio-Rad; Hercules, California). The reaction conditions were the following: 98 °C 30 sec, (98 °C 3 sec, 60,4 °C 20 sec, 72 °C 10 sec)x39cycles. In order to confirm the specificity of the reaction, a melting curve was performed after each PCR (from 65 °C to 95 °C with an increase of temperature of 0.5 °C/sec). The annealing temperature was optimized by performing a gradient with each primer pair (from from 55 °C to 64 °C). All reactions were performed in duplicate; the raw values were calculated using three housekeeping genes as reference (GAPDH, SDHA, PBGD) according to the ΔΔCq method used by the CFX Manager Software (BioRad).

## Electronic supplementary material

Supplementary Files
