# Supplementary material for: Ensemble Modeling Approach Targeting Heterogeneous RNA-Seq data: Application to Melanoma Pseudogenes
Source: Sci Rep. 2017 Dec 11;7:17344. doi: 10.1038/s41598-017-17337-7 (PMC5725464; doi:10.1038/s41598-017-17337-7)
Supplement: Supplementary file 1 — Supplementary Files [file 41598_2017_17337_MOESM1_ESM.zip › Supplementary_Files/7_Gene_Families/Supplementary Table 7.1.pdf]

Gene Families

Individual Reports

Intronic Read Counts

DESeq

	cytokines and growth factors	transcription factors	homeodomain proteins	cell differentiation markers	protein kinases	translocated cancer genes	oncogenes	tumor suppressors
tumor suppressors	0	2	0	0	0	0	0	7
oncogenes	3	29	8	11	11	49	60	
translocated cancer genes	3	24	8	7	9	50		
protein kinases	2	1	0	8	89			
cell differentiation markers	12	0	0	134				
homeodomain proteins	0	74	74					
transcription factors	0	243						
cytokines and growth factors	161							

Intronic Read Counts

NOISeq

	cytokines and growth factors	transcription factors	homeodomain proteins	cell differentiation markers	protein kinases	translocated cancer genes	oncogenes	tumor suppressors
**tumor suppressors**	0	0	0	0	0	0	0	0
**oncogenes**	1	1	1	2	1	4	4	
**translocated cancer genes**	1	1	1	2	1	4		
**protein kinases**	1	0	0	1	4			
**cell differentiation markers**	1	0	0	18				
**homeodomain proteins**	0	1	1					
**transcription factors**	0	5						
**cytokines and growth factors**	18							

Exonic Read Counts

DESeq

	cytokines and growth factors	transcription factors	homeodomain proteins	cell differentiation markers	protein kinases	translocated cancer genes	oncogenes	tumor suppressors
**tumor suppressors**	0	1	0	0	0	0	0	4
**oncogenes**	3	23	7	10	9	47	52	
**translocated cancer genes**	3	21	7	8	8	48		
**protein kinases**	2	1	0	7	70			
**cell differentiation markers**	11	0	0	112				
**homeodomain proteins**	0	48	48					
**transcription factors**	0	179						
**cytokines and growth factors**	127							

Exonic Read Counts

NOISeq

	cytokines and growth factors	transcription factors	homeodomain proteins	cell differentiation markers	protein kinases	translocated cancer genes	oncogenes	tumor suppressors
**tumor suppressors**	0	0	0	0	0	0	0	0
**oncogenes**	1	1	1	2	1	5	5	
**translocated cancer genes**	1	1	1	2	1	5		
**protein kinases**	1	0	0	2	7			
**cell differentiation markers**	1	0	0	27				
**homeodomain proteins**	0	2	2					
**transcription factors**	0	11						
**cytokines and growth factors**	25							

CuffDiff

	cytokines and growth factors	transcription factors	homeodomain proteins	cell differentiation markers	protein kinases	translocated cancer genes	oncogenes	tumor suppressors
**tumor suppressors**	0	1	0	0	0	0	0	8
**oncogenes**	2	16	4	9	10	52	57	
**translocated cancer genes**	2	16	4	6	9	54		
**protein kinases**	1	2	0	5	79			
**cell differentiation markers**	3	0	0	94				
**homeodomain proteins**	0	18	18					
**transcription factors**	0	127						
**cytokines and growth factors**	83							

FPKM

Limma

	cytokines and growth factors	transcription factors	homeodomain proteins	cell differentiation markers	protein kinases	translocated cancer genes	oncogenes	tumor suppressors
**tumor suppressors**	0	2	0	0	1	0	0	9
**oncogenes**	2	23	5	9	11	56	65	
**translocated cancer genes**	2	21	5	5	9	59		
**protein kinases**	1	2	0	7	101			
**cell differentiation markers**	5	0	0	105				
**homeodomain proteins**	0	36	36					
**transcription factors**	0	176						
**cytokines and growth factors**	83							

FPKM

GeneSpring

	cytokines and growth factors	transcription factors	homeodomain proteins	cell differentiation markers	protein kinases	translocated cancer genes	oncogenes	tumor suppressors
**tumor suppressors**	0	1	0	0	0	0	0	6
**oncogenes**	0	15	2	4	7	49	56	
**translocated cancer genes**	0	14	2	2	6	51		
**protein kinases**	0	2	0	3	82			
**cell differentiation markers**	0	0	0	45				
**homeodomain proteins**	0	7	7					
**transcription factors**	0	163						
**cytokines and growth factors**	26							
