# Supplementary material for: Ensemble Modeling Approach Targeting Heterogeneous RNA-Seq data: Application to Melanoma Pseudogenes
Source: Sci Rep. 2017 Dec 11;7:17344. doi: 10.1038/s41598-017-17337-7 (PMC5725464; doi:10.1038/s41598-017-17337-7)
Supplement: Supplementary file 1 — Supplementary Files [file 41598_2017_17337_MOESM1_ESM.zip › Supplementary_Files/9_Validations/Supplementary Fig. 9.1.pptx]

## Slide 1
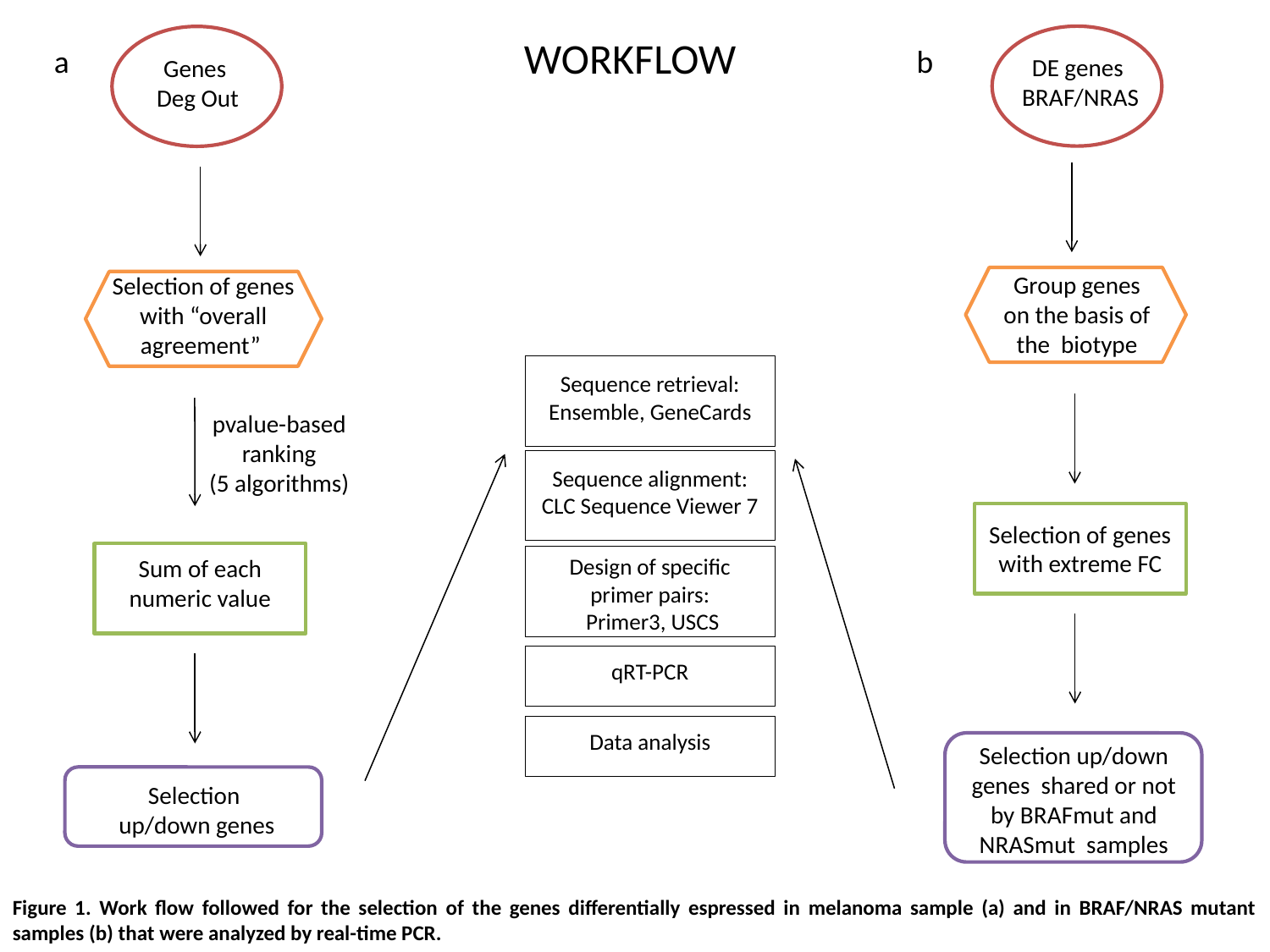

# WORKFLOW
DE genes
 BRAF/NRAS
Selection of genes with extreme FC
Selection up/down genes shared or not by BRAFmut and NRASmut samples
Group genes
on the basis of the biotype
Genes
Deg Out
Sum of each numeric value
Selection
up/down genes
Selection of genes with “overall agreement”
pvalue-based
ranking
(5 algorithms)
a
b
Sequence retrieval:
Ensemble, GeneCards
Sequence alignment:
CLC Sequence Viewer 7
Design of specific primer pairs:
 Primer3, USCS
qRT-PCR
Data analysis
Figure 1. Work flow followed for the selection of the genes differentially espressed in melanoma sample (a) and in BRAF/NRAS mutant samples (b) that were analyzed by real-time PCR.

## Slide 2
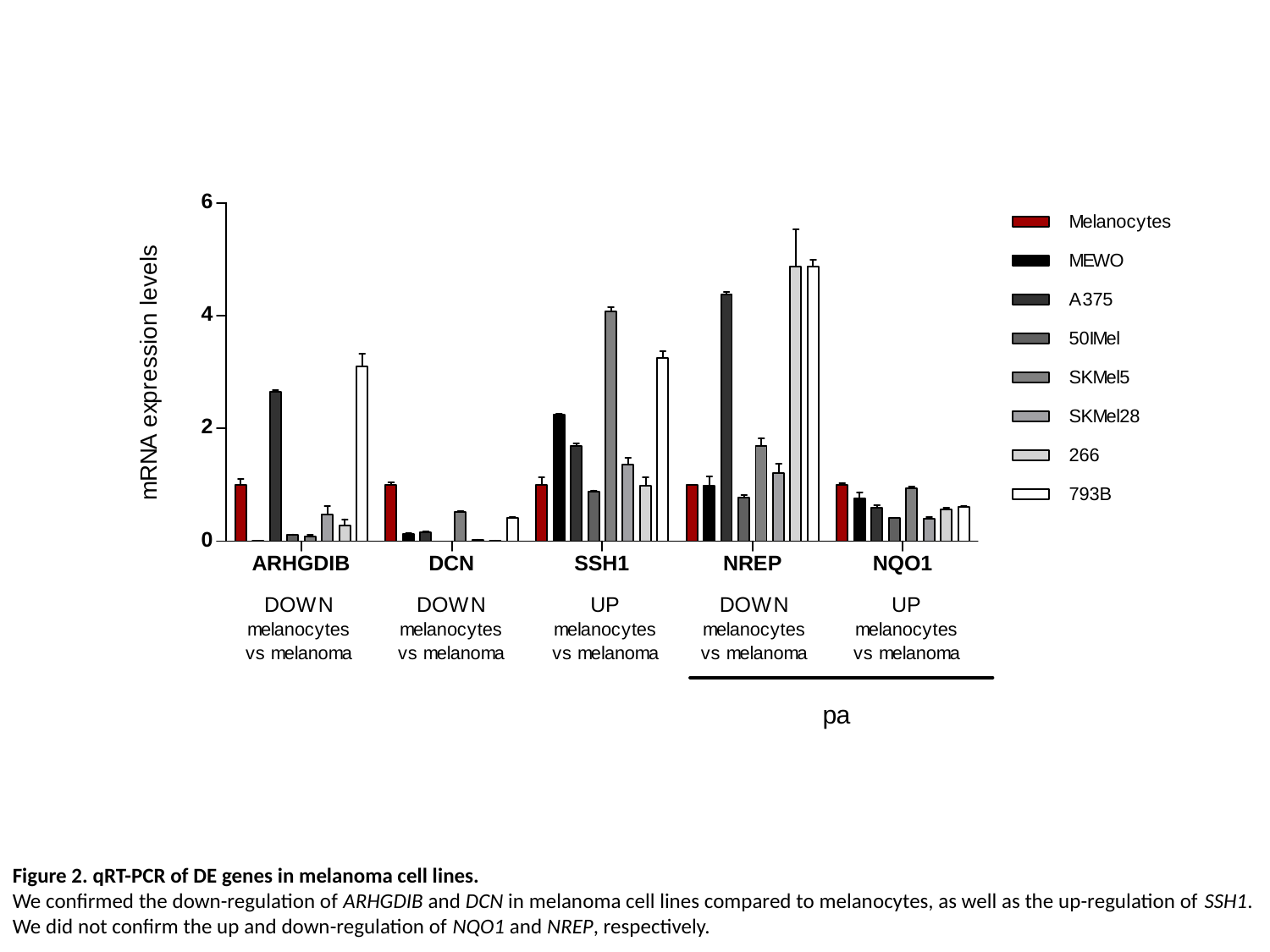

Figure 2. qRT-PCR of DE genes in melanoma cell lines.
We confirmed the down-regulation of ARHGDIB and DCN in melanoma cell lines compared to melanocytes, as well as the up-regulation of SSH1. We did not confirm the up and down-regulation of NQO1 and NREP, respectively.

## Slide 3
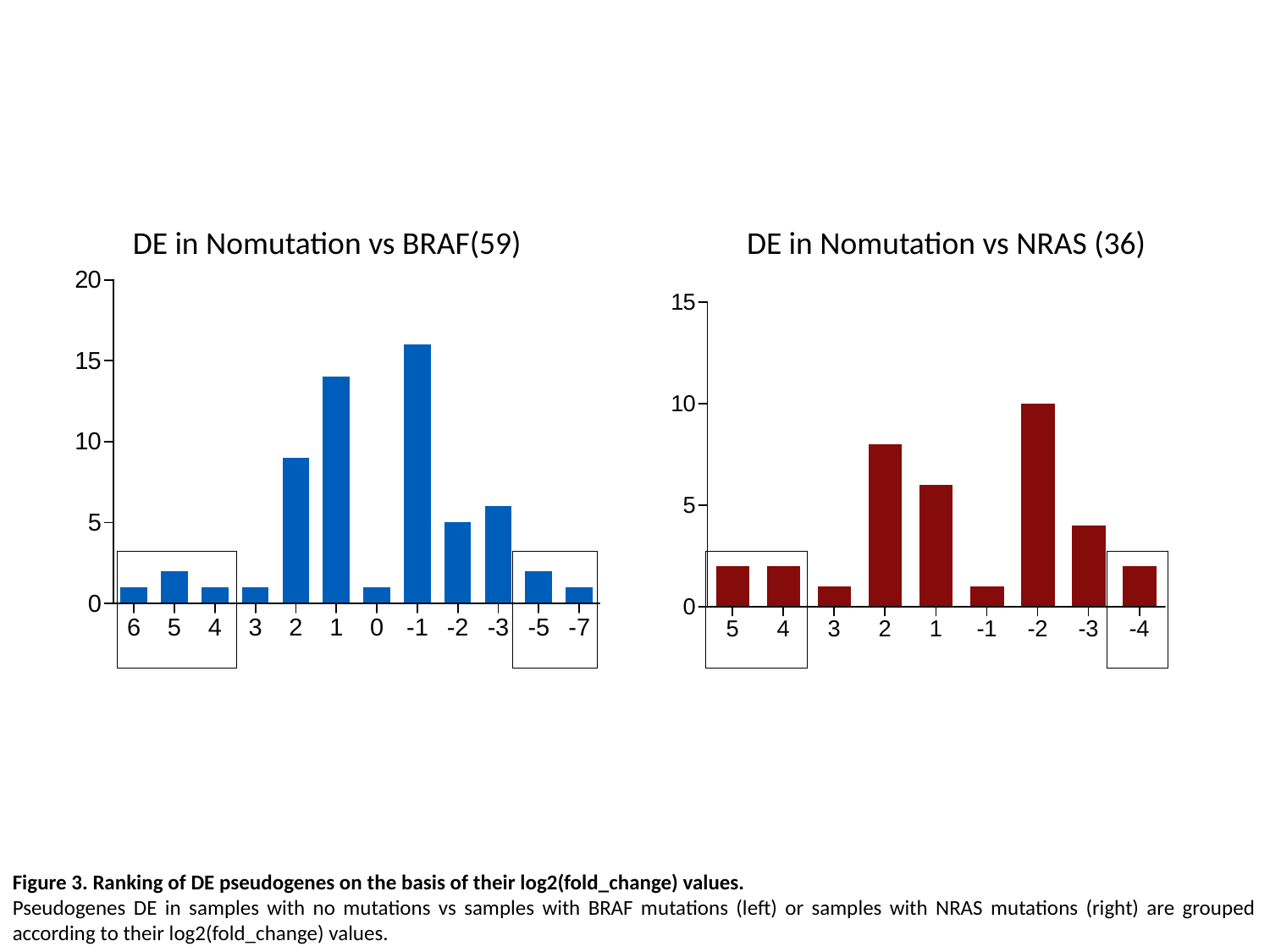

DE in Nomutation vs BRAF(59)
DE in Nomutation vs NRAS (36)
Figure 3. Ranking of DE pseudogenes on the basis of their log2(fold_change) values.
Pseudogenes DE in samples with no mutations vs samples with BRAF mutations (left) or samples with NRAS mutations (right) are grouped according to their log2(fold_change) values.

## Slide 4
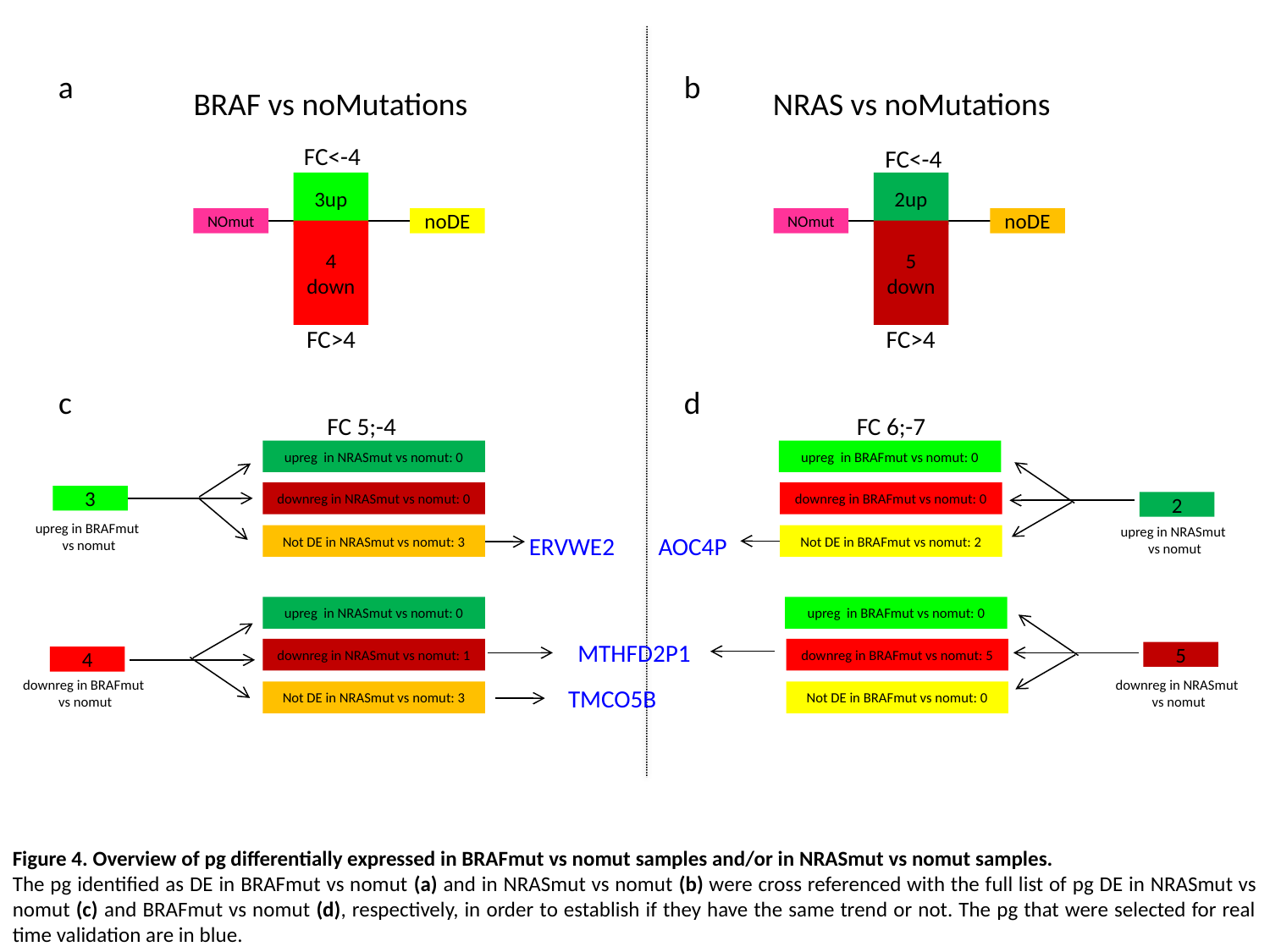

BRAF vs noMutations
NRAS vs noMutations
FC<-4
FC<-4
3up
2up
NOmut
noDE
NOmut
noDE
4
down
5
down
FC>4
FC>4
FC 5;-4
FC 6;-7
upreg in NRASmut vs nomut: 0
downreg in NRASmut vs nomut: 0
3
upreg in BRAFmut
vs nomut
Not DE in NRASmut vs nomut: 3
upreg in BRAFmut vs nomut: 0
downreg in BRAFmut vs nomut: 0
2
Not DE in BRAFmut vs nomut: 2
upreg in NRASmut
vs nomut
ERVWE2
AOC4P
upreg in NRASmut vs nomut: 0
downreg in NRASmut vs nomut: 1
Not DE in NRASmut vs nomut: 3
upreg in BRAFmut vs nomut: 0
downreg in BRAFmut vs nomut: 5
Not DE in BRAFmut vs nomut: 0
5
4
MTHFD2P1
downreg in BRAFmut
vs nomut
downreg in NRASmut
vs nomut
TMCO5B
a
b
c
d
Figure 4. Overview of pg differentially expressed in BRAFmut vs nomut samples and/or in NRASmut vs nomut samples.
The pg identified as DE in BRAFmut vs nomut (a) and in NRASmut vs nomut (b) were cross referenced with the full list of pg DE in NRASmut vs nomut (c) and BRAFmut vs nomut (d), respectively, in order to establish if they have the same trend or not. The pg that were selected for real time validation are in blue.

## Slide 5
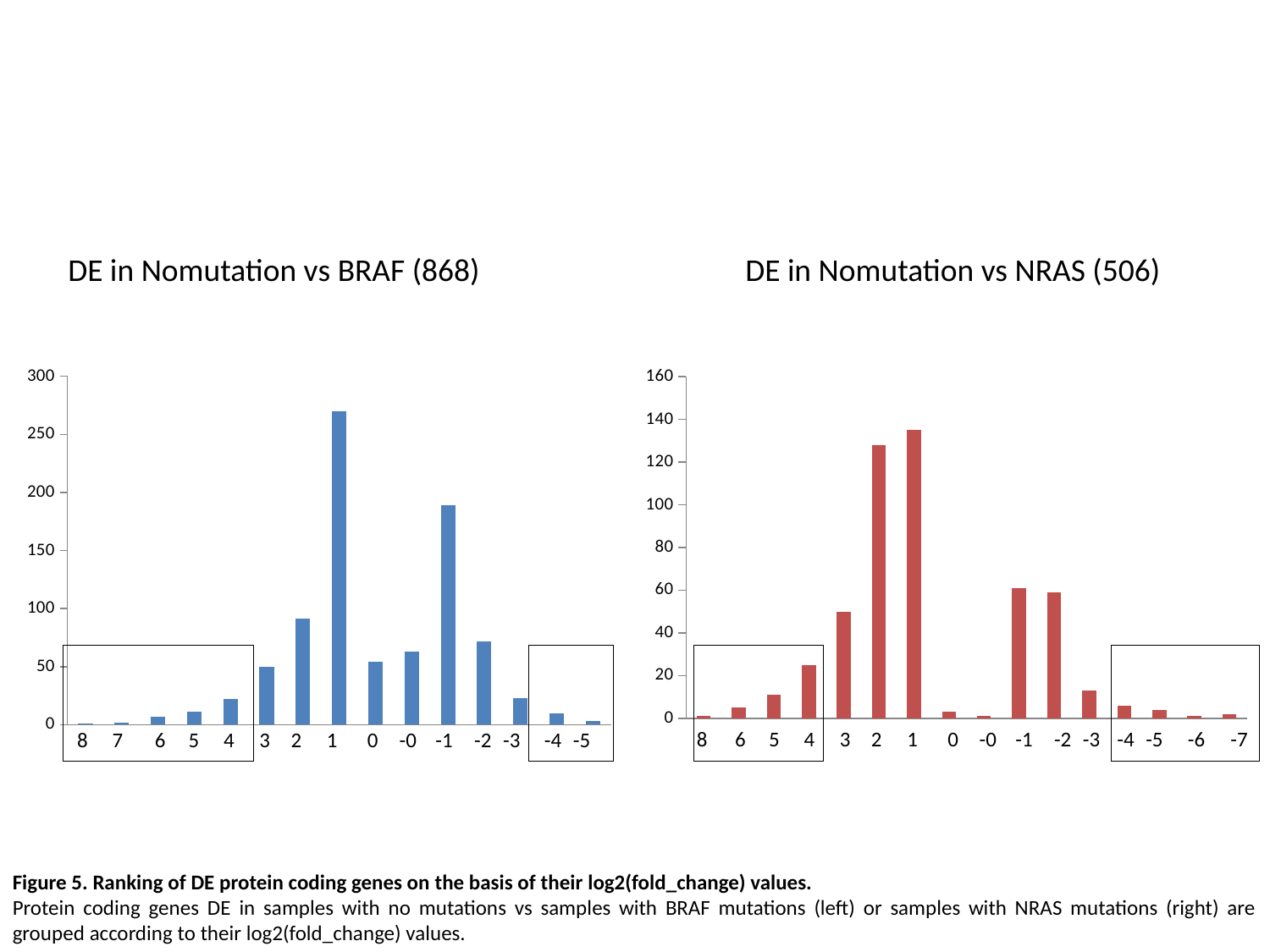

DE in Nomutation vs BRAF (868)
DE in Nomutation vs NRAS (506)
### Chart
| Category | |
|---|---|8
7
6
5
4
3
2
1
0
-0
-1
-2
-3
-4
-5
### Chart
| Category | |
|---|---|8
6
5
4
3
2
1
0
-0
-1
-2
-3
-4
-5
-6
-7
Figure 5. Ranking of DE protein coding genes on the basis of their log2(fold_change) values.
Protein coding genes DE in samples with no mutations vs samples with BRAF mutations (left) or samples with NRAS mutations (right) are grouped according to their log2(fold_change) values.

## Slide 6
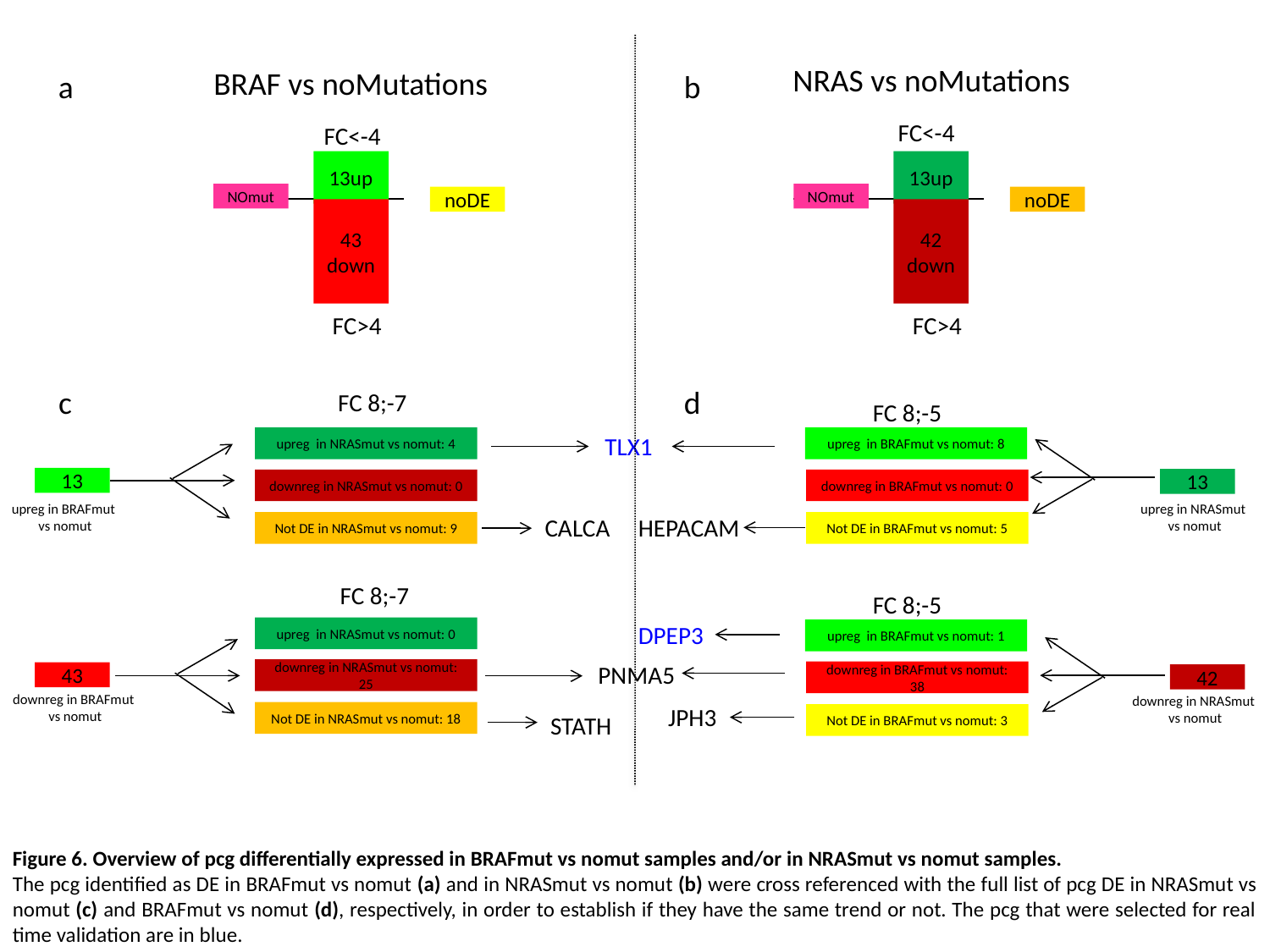

NRAS vs noMutations
BRAF vs noMutations
FC<-4
FC<-4
13up
13up
NOmut
NOmut
noDE
noDE
43
down
42
down
FC>4
FC>4
FC 8;-7
FC 8;-5
TLX1
upreg in NRASmut vs nomut: 4
13
downreg in NRASmut vs nomut: 0
Not DE in NRASmut vs nomut: 9
upreg in BRAFmut vs nomut: 8
13
downreg in BRAFmut vs nomut: 0
Not DE in BRAFmut vs nomut: 5
upreg in BRAFmut
vs nomut
upreg in NRASmut
vs nomut
CALCA
HEPACAM
FC 8;-7
upreg in NRASmut vs nomut: 0
43
downreg in NRASmut vs nomut: 25
Not DE in NRASmut vs nomut: 18
FC 8;-5
DPEP3
upreg in BRAFmut vs nomut: 1
42
downreg in BRAFmut vs nomut: 38
Not DE in BRAFmut vs nomut: 3
PNMA5
downreg in BRAFmut
vs nomut
downreg in NRASmut
vs nomut
JPH3
STATH
a
b
c
d
Figure 6. Overview of pcg differentially expressed in BRAFmut vs nomut samples and/or in NRASmut vs nomut samples.
The pcg identified as DE in BRAFmut vs nomut (a) and in NRASmut vs nomut (b) were cross referenced with the full list of pcg DE in NRASmut vs nomut (c) and BRAFmut vs nomut (d), respectively, in order to establish if they have the same trend or not. The pcg that were selected for real time validation are in blue.

## Slide 7
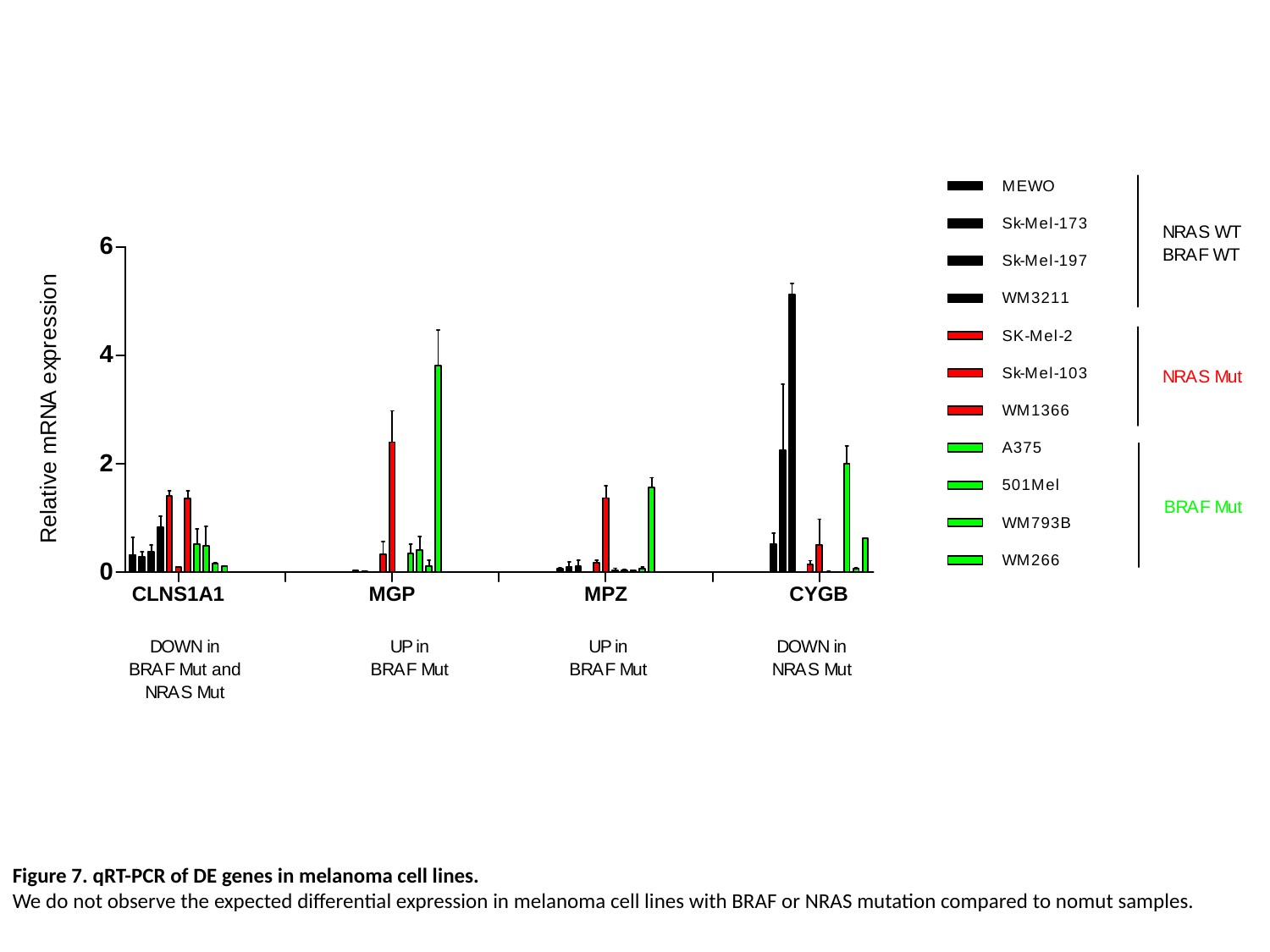

Figure 7. qRT-PCR of DE genes in melanoma cell lines.
We do not observe the expected differential expression in melanoma cell lines with BRAF or NRAS mutation compared to nomut samples.

## Slide 8
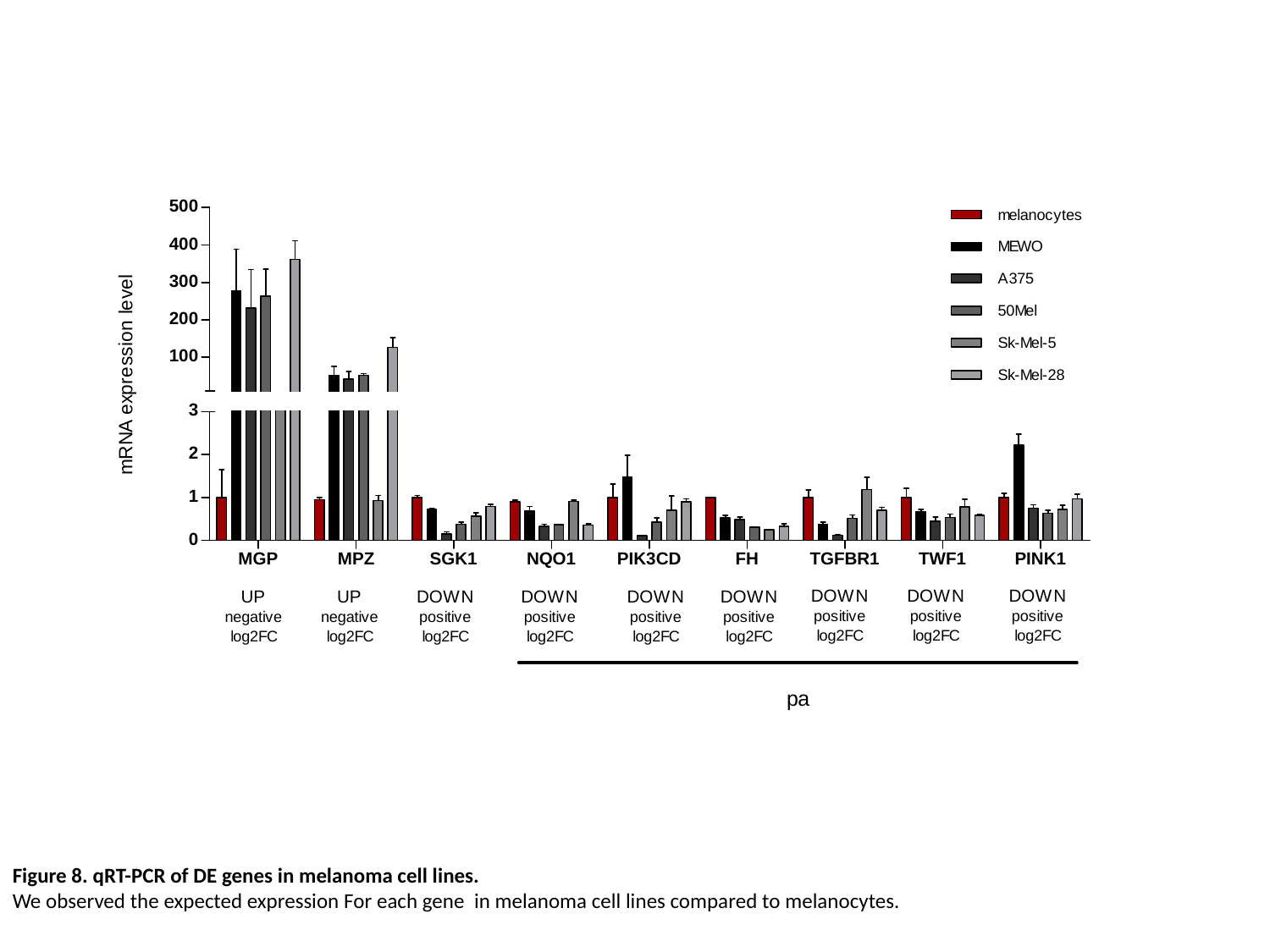

Figure 8. qRT-PCR of DE genes in melanoma cell lines.
We observed the expected expression For each gene in melanoma cell lines compared to melanocytes.
