# Supplementary material for: Ensemble Modeling Approach Targeting Heterogeneous RNA-Seq data: Application to Melanoma Pseudogenes
Source: Sci Rep. 2017 Dec 11;7:17344. doi: 10.1038/s41598-017-17337-7 (PMC5725464; doi:10.1038/s41598-017-17337-7)
Supplement: Supplementary file 1 — Supplementary Files [file 41598_2017_17337_MOESM1_ESM.zip › Supplementary_Files/9_Validations/Supplementary Text 9.2.docx]

**Validation Results**

**Work Flow for the selection of the genes differentially expressed in BRAF/NRAS mutant samples that were selected for validation by real-time PCR.**

The main steps of the work flow are summarized in Figure 1b.

Starting from the tables “CuffDiff DE gene BRAF” (Table 9) and “CuffDiff DE gene NRAS” (Table 10), in which the coding and non-coding genes differentially expressed (DE) between melanoma samples without mutation in BRAF/NRAS and melanoma samples with BRAF or NRAS mutation are reported, we focused our attention on DE pseudogenes (pg) and protein coding genes (pcg) (see Table 11 and Table 12 for the total number of genes belonging to each group).

We ranked the pg on the basis of their log2(fold_change) values, obtaining the distributions that are reported in Fig. 3. For further analysis, we decided to consider only those pg with extreme fold changes. Specifically, we focused on those that display log2(fold_change) values higher than 4 and lower than -4. In this way, we ended up with 3 up- and 4 down-regulated pg from the BRAF list (Fig. 4a), as well as 2 up- and 5 down-regulated pg from the NRAS list (Fig. 4b). The pg belonging to these 2 shortlists were cross referenced, in order to establish if, irrespectively of the fold change, they are differentially expressed only in BRAFmut samples, only in NRASmut samples or in both. The spectrum of cases obtained is summarized in Fig. 4c-d: the 3 pg up-regulated in BRAFmut vs nomut are not DE in NRASmut vs nomut; among the 4 pg down-regulated in BRAFmut vs nomut, one of them is also down-regulated in NRASmut vs nomut, while the other 3 are not DE; the 2 pg up-regulated in NRASmut vs nomut are not DE in BRAFmut vs nomut; one of the 5 pg down-regulated in NRASmut vs nomut is also down-regulated in BRAFmut vs nomut. For validation by real-time PCR, we picked 4 pg for which it was possible to design specific primers: ERVWE2, a pg up-regulated only in BRAFmut vs nomut samples; TMCO5B, a pg down-regulated only in BRAFmut vs nomut samples; AOC4P, the pg up-regulated only in NRASmut vs nomut samples; MTHFD2P1, a pg down-regulated in both groups. The same analysis was applied to the pcg. By ranking them on the basis of their log2(fold_change) values, we obtained the distributions that are reported in Fig. 5. Again, we considered only those genes with extreme fold changes (higher than 4 and lower than -4). We selected 13 up- and 43 down-regulated pcg from the BRAF list (Fig. 6a), as well as 13 up- and 42 down-regulated pcg from the NRAS list (Fig. 6b). The pcg belonging to these shortlists were cross referenced to verify if they are differentially expressed only in BRAFmut samples, only in NRASmut samples or in both. The outcome is show in Fig. 6c-d. For validation by real-time PCR we picked 2 pcg, DPEP3 that is down-regulated in NRASmut vs nomut and up-regulated in BRAFmut and TLX1 that is up-regulated in BRAFmut and NRASmut. Unfortunately, all the selected pg and pcg resulted undetectable according to qRT-PCR. Therefore, we decided to change selection strategy.

We ranked pseudogenes and protein coding genes on the basis of value-1 and value-2, which are a measure of their expression levels in BRAFmut or NRASmut samples and nomut samples, respectively. All the DE pseudogenes show low values and have not been considered for validation. After that protein coding genes from BRAFmut and NRASmut tables were ranked on the basis of the two values (Table 13a, b; Table 14a,b), those with the highest values were scanned looking for candidates for which it was possible design specific primer pairs. We ended up with MPZ and MGP that are up-regulated in BRAFmut vs nomut, with CLN1A1 that is down-regulated in both BRAFmut and NRASmut vs nomut and with CYBC that is down-regulated in NRASmut vs nomut samples. We compared their expression levels in melanoma cell lines harboring BRAF or NRAS mutation with melanoma cell lines without mutations and, unfortunately, we did not confirm the trend shown in melanoma samples (Fig. 7).
