# Supplementary material for: Ensemble Modeling Approach Targeting Heterogeneous RNA-Seq data: Application to Melanoma Pseudogenes
Source: Sci Rep. 2017 Dec 11;7:17344. doi: 10.1038/s41598-017-17337-7 (PMC5725464; doi:10.1038/s41598-017-17337-7)
Supplement: Supplementary file 1 — Supplementary Files [file 41598_2017_17337_MOESM1_ESM.zip › Supplementary_Files/9_Validations/Supplementary Text 9.3.docx]

**qRT-PCR Primers**

| Primer qRT-PCR | Sequence | Tm |
| --- | --- | --- |
| *ARHGDIB Fw* | TGACGATGATGAGCTGGACA | 60,4 |
| *ARHGDIB Rev* | TGTCACCACAGGACCATCTC |  |
| *SSH1 Fw* | TGATCAACCTTCTGCGTTGC |  |
| *SSH1 Rev* | AAAGTCCACTCCCAGCAAGA |  |
| *NREP Fw* | TTGTCTGTTGGTCTCCCTGG |  |
| *NREP Rev* | CCTTCCCTCCATGTCCTTGT |  |
| *DCN Fw* | ATGAGGCTTCTGGGATAGGC |  |
| *DCN Rev* | CAGAACACTGGACCACTCGA |  |

**Table 1:** Primers used in qRT-PCR to detect DE genes in melanoma cell lines compared to melanocytes.

| Primer qRT-PCR | Sequence | Tm |
| --- | --- | --- |
| *TMCO5B Fw* | GTCCCCGAGTCCCTAAGAAC | 60,4 |
| *TMCO5B Rev* | ATCTTGGCCAGTTCCTCCTC |  |
| *ERVWE2 Fw* | TCCAATCCCACCATCTGCTT |  |
| *ERVWE2 Rev* | TGTGTTGGAGTTTCTTGGCG |  |
| *MTHFD2P1 Fw* | ACAGCACGGCATTTTGGAAT |  |
| *MTHFD2P1 Rev* | GAAGAGGCAGCTGAACAAGG |  |
| *AOC4P Fw* | TCCGTGGAGAGAGGCTTCA |  |
| *AOC4P Rev* | GGGTGTCCAAGGGTCATTCA |  |
| *DPEP3 Fw* | AACTCGAGCTTGTGACCTCA |  |
| *DPEP3 Rev* | AGCACATAGAAACTGCGCAG |  |
| *TLX1 Fw* | GCCTATGGTACTGGAGGTCC |  |
| *TLX1 Rev* | GGCCATGTTCACGTTGTAGG |  |

**Table 2:** Primers used to detect pg and pcg differentially expressed in BRAFmut and NRASmut samples and prioritized on the basis of their Fc values.

| Primer qRT-PCR | Sequence | Tm |
| --- | --- | --- |
| *CYGB Fw* | CCAGTTCAAGCACATGGAGG | 60,4 |
| *CYGB Rev* | GGGGTCATGCAGGTTCTCC |  |
| *CLNS1A Fw* | CTCGGCACTGGTACCCTTTA |  |
| *CLNS1A Rev* | TTCGGTCCCTGGATAATGCA |  |
| *MPZ Fw* | GAACTCGGACAAGTGATCGC |  |
| *MPZ Rev* | GAGCAAAGAGGGAAAGCACC |  |
| *MGP Fw* | GTCCACGAGCTCAATAGGGA |  |
| *MGP Rev* | AGTCTCATTTGGTCCCTCGG |  |

**Table 3:** Primers used to detect pg and pcg differentially expressed in BRAFmut and NRASmut samples and prioritized on the basis of their expression values.

| Primer qRT-PCR | Sequence | Tm |
| --- | --- | --- |
| *ATPA1 Fw* | CTCAGATGTGTCCAAGCAA | 57 |
| *ATPA1 Rev* | GTCAGTGCCCAAGTCAATG |  |
| *GAPDH Fw* | CGCTCTCTGCTCCTCCTGTT | 60,4 |
| *GAPDH Rer* | CCATGGTGTCTGAGCGATGT |  |
| *PBGD Fow* | TCCAAGCGGAGCCATGTCTG |  |
| *PBGD Rev* | AGAATCTTGTCCCCTGTGGTGGA |  |
| *SDHA Fw* | CCACTCGCTATTGCACACC |  |
| *SDHA Rev* | CACTCCCCATTCTCCATCA |  |

**Table 4:** Primers used to detect gDNA contamination (ATPA1) and as housekeeping genes (GAPDH, PBGD and SDHA).

| **Primer qRT-PCR** | **Sequence** | **Tm** |
| --- | --- | --- |
| **NQO1 Fw** | AGAAAGGATGGGAGGTGGTG | 60,4 |
| **NQO1 Rev** | GAAAGTTCGCAGGGTCCTTC |  |
| **TGFBR1 Fw** | GGAACTGGCAGCTGTCATTG |  |
| **TGFBR1 Rev** | AGGGTCCTCTTCATTTGGCA |  |
| **TWF1 Fw** | TGGAGGACAAACAACCATGC |  |
| **TWF1 Rev** | AGTTGCTCTTGTTGCTGCAT |  |
| **PINK1 Fw** | ATCCAAGAGAGGTCCCAAGC |  |
| **PINK1 Rev** | AGCACATCAGGGTAGTCGAC |  |
| **SGK1 Fw** | ATGAAGCAGAGGAGGATGGG |  |
| **SGK1 Rev** | AGGAGAAGGGTTGGCATTCA |  |
| **PI3 Fw** | GTCAAAGGCCGTGTTCCATT |  |
| **PI3 Rev** | TGGACCTTTGACTGGCTCTT |  |
| **PIK3CD Fw** | GAAACTGACGGACGATGAGC |  |
| **PIK3CD Rev** | GGTGCCAGAAAAGGAAGTGG |  |
| **SFN Fw** | GGGAGAAGGTGGAGACTGAG |  |
| **SFN Rev** | GGTAGCGGTAGTAGTCACCC |  |
| **FH Fw** | GCACTTACAGGCTTGCCTTT |  |
| **FH Rev** | AGGCAGTAGTGTTCATGGCT |  |

**Table 5:** Primers used to identify pcg and pa deregulated pathway between melanocytes and melanoma samples.
