# Supplementary material for: Ensemble Modeling Approach Targeting Heterogeneous RNA-Seq data: Application to Melanoma Pseudogenes
Source: Sci Rep. 2017 Dec 11;7:17344. doi: 10.1038/s41598-017-17337-7 (PMC5725464; doi:10.1038/s41598-017-17337-7)
Supplement: Supplementary file 1 — Supplementary Files [file 41598_2017_17337_MOESM1_ESM.zip › Supplementary_Files/Title page and outline.pdf]

Ensemble Modeling Approach Targeting Heterogeneous RNA-Seq data: Application to Melanoma Pseudogenes.

Enrico Capobianco,*[1] Camilo Valdes,[1] Samanta Sarti,[2] Zhijie Jiang,[1] Laura Poliseno,[3] Nicolas F. Tsinoremas[1,4]

[1]Center for Computational Science, University of Miami, Miami, FL, USA

[2] University of Siena, Italy

[3] Istituto Toscano Tumori Oncogenomics Unit, Institute of Clinical Physiology - National Research Council, Pisa, Italy

[4] Department of Medicine, Miller School of Medicine, University of Miami, Miami, FL, USA

*****corresponding author**:** **ecapobianco@med.miami.edu**

SUPPLEMENTARY MATERIALS

Supplementary Materials

Contents

Each line item in the list below is a **folder**, and each nested item is a **nested folder**.

1. **BLAST**
   - 1.1. Supplementary Table 1.1.xlsx *(blast results)*
   - 1.2. Supplementary Table 1.2.txt *(parental IDs)*
2. **Quality Control Quality Assurance**
   - **2.1. Counts**
     - 2.1.1. Supplementary Table 2.1.1.txt *(exonic counts)*
     - 2.1.2. Supplementary Table 2.1.2.txt *(intronic counts)*
   - **2.2. FPKM**
     - 2.2.1. Supplementary Table 2.2.1.txt *(fpkm)*
3. **Methods**
   - **3.1. DESeq**
     - 3.1.1. Supplementary Table 3.1.1.txt *(deseq results)*
   - **3.2. NOISeq**
     - **3.2.1. NOISeq trials**
       - 3.2.1.1. Supplementary Table 3.2.1.1.txt *(q=0.60)*
       - 3.2.1.2. Supplementary Table 3.2.1.2.txt *(q=0.65)*
       - 3.2.1.3. Supplementary Table 3.2.1.3.txt *(q=0.70)*
       - 3.2.1.4. Supplementary Table 3.2.1.4.txt *(q=0.75)*
       - 3.2.1.5. Supplementary Table 3.2.1.5.txt *(q=0.80)*
       - 3.2.1.6. Supplementary Table 3.2.1.6.txt *(q=0.85)*
     - 3.2.2. Supplementary Table 3.2.2.txt *(noiseq results)*
   - **3.3. CuffDiff**
     - 3.3.1. Supplementary Table 3.3.1.txt (cuffdiff results)
   - **3.4. Limma**
     - 3.4.1. Supplementary Table 3.4.1.txt (limma results)
   - **3.5. GeneSpring**
     - 3.5.1. Supplementary Table 3.5.1.txt (genespring results)
4. **Sample Driven Gene Detection Sensitivity Analysis**
   - 4.1. Supplementary Fig. 4.1.png *(counts filtering)*
   - 4.2. Supplementary Fig. 4.2.png *(fpkm filtering)*
5. **Cross-Evidences from Expression Profiling**
   - **5.1. Differentially Expressed Genes (DEGs)**
     - **5.1.1. DESeq**

5.1.1.1. Supplementary Table 5.1.1.1.txt *(deseq degs)*

5.1.2. NOISeq

5.1.2.1. Supplementary Table 5.1.2.1.txt *(noiseq degs)*

5.1.3. CuffDiff

5.1.3.1. Supplementary Table 5.1.3.1.txt *(cuffdiff degs)*

5.1.4. Limma

5.1.4.1. Supplementary Table 5.1.4.1.txt *(limma degs)*

5.1.5. GeneSpring

5.1.5.1. Supplementary Table 5.1.5.1.txt *(genespring degs)*

5.2. Biotype Decomposition

5.2.1. Supplementary Fig. 5.2.1.png *(all biotypes)*

5.3. Consensus Parental Gene Pseudogene Associations

5.3.1. Supplementary Table 5.3.1.xlsx *(parental genes)*

5.3.2. Supplementary Table 5.3.2.xlsx *(pseudogenes)*

5.3.3. Supplementary Fig. 5.3.3.png *(parental gene annotations)*

5.3.4. Supplementary Fig. 5.3.4.png *(pseudogene annotations)*

5.4. Intronic Evidences

5.4.1. DESeq

5.4.1.1. Supplementary Table 5.4.1.1.txt *(deseq Intronic counts)*

5.4.2. NOISeq

5.4.2.1. Supplementary Table 5.4.2.1.txt *(noiseq Intronic counts)*

6. Bio-Annotations

6.1. Core Consensus Annotations

6.1.1. Pathways

6.1.1.1. DESeq

6.1.1.1.1. Supplementary Fig. 6.1.1.1.1.xlsx *(deseq pathways)*

6.1.1.2. NOISeq

6.1.1.2.1. Supplementary Fig. 6.1.1.2.1.xlsx *(noiseq pathways)*

6.1.1.3. CuffDiff

6.1.1.3.1. Supplementary Fig. 6.1.1.3.1.xlsx *(cuffdiff pathways)*

6.1.1.4. Limma

6.1.1.4.1. Supplementary Fig. 6.1.1.4.1.xlsx *(limma pathways)*

6.1.1.5. GeneSpring

6.1.1.5.1. Supplementary Fig. 6.1.1.5.1.xlsx *(genespring pathways)*

6.1.2. GO

6.1.2.1. DESeq

6.1.2.1.1. Supplementary Fig. 6.1.2.1.1.xlsx *(deseq GO)*

6.1.2.2. NOISeq

6.1.2.2.1. Supplementary Fig. 6.1.2.2.1.xlsx *(noiseq GO)*

6.1.2.3. CuffDiff

6.1.2.3.1. Supplementary Fig. 6.1.2.3.1.xlsx *(cuffdiff GO)*

6.1.2.4. Limma

6.1.2.4.1. Supplementary Fig. 6.1.2.4.1.xlsx *(limma GO)*

6.1.2.5. GeneSpring

6.1.2.5.1. Supplementary Fig. 6.1.2.5.1.xlsx *(genespring GO)*

6.2. Ensembl Annotations Comparison

6.2.1. Supplementary Table 6.2.1.txt *(ensembl v.72)*

6.2.2. Supplementary Table 6.2.2.txt *(ensembl v.86)*

6.2.3. Supplementary Table 6.2.3.xlsx *(comparison file 72 vs 86)*

6.2.4. Supplementary Fig. 6.2.4.png *(Ensembl frequency)*

7. Gene Families

7.1. Supplementary Table 7.1.pdf *(consensus gene families)*

8. Models

8.1. PCA Gene Selection

8.1.1. Supplementary Table 8.1.1.txt *(pca deseq results)*

8.1.2. Supplementary Table 8.1.2.txt *(pca noiseq results)*

8.1.3. Supplementary Table 8.1.3.txt *(pca cuffdiff results)*

8.1.4. Supplementary Table 8.1.4.txt *(pca limma results)*

8.1.5. Supplementary Table 8.1.5.txt *(pca genespring results)*

8.1.6. Supplementary Fig. 8.1.1.png *(pca selections)*

8.1.7. Supplementary Fig. 8.1.2.png *(box plot selection)*

8.2. Linear Regression Model (LRM) Selection

8.2.1. Supplementary Table 8.2.1.txt *(lrm deseq as response)*

8.2.2. Supplementary Table 8.2.2.txt *(lrm noiseq as response)*

8.2.3. Supplementary Table 8.2.3.txt *(lrm cuffdiff as response)*

8.2.4. Supplementary Table 8.2.4.txt *(lrm genespring as response)*

8.2.5. Supplementary Table 8.2.5.xlsx *(lrm average responses for input into IQR)*

8.2.6. Supplementary Table 8.2.6.txt *(lrm results @ IQR=2)*

8.2.7. Supplementary Table 8.2.7.txt *(lrm results @ IQR=2.5)*

8.2.8. Supplementary Table 8.2.8.txt *(lrm results @ IQR=3)*

8.2.9. Supplementary Fig. 8.2.9.png *(lrm selections plot)*

8.2.10. Supplementary Fig. 8.2.10.png *(lrm coefficient plot)*

8.3. PCA-LRM Results Intersection

8.3.1. Supplementary Fig. 8.3.1.png *(pca & lrm venn)*

8.4. PCA-LRM Pathways

8.4.1. Supplementary Fig. 8.4.1.png *(pathway overview)*

8.4.2. IQR at 2

8.4.2.1. Supplementary Table 8.4.2.1.xlsx *(iqr 2 pathways)*

8.4.3. IQR at 2.5

8.4.3.1. Supplementary Table 8.4.3.1.xlsx *(iqr 2.5 pathways)*

8.4.4. IQR at 3

8.4.4.1. Supplementary Table 8.4.4.1.xlsx *(iqr 3 pathways)*

8.5. PCA-LRM Gene Families

8.5.1. Supplementary Fig. 8.5.1.png *(gene families)*

8.6. Parental Gene – Pseudogene Associations

8.6.1. Supplementary Fig. 8.6.1.png *(needle plot)*

8.6.2. Supplementary Fig. 8.6.2.png *(small network)*

8.6.3. Supplementary Fig. 8.6.3.png *(small network, clean)*

8.6.4. Supplementary Fig. 8.6.4.png *(big network)*

8.6.5. Supplementary Table 8.6.5.xlsx *(associations table)*

9. Validations

9.1. Supplementary Fig. 9.1.pptx *(validation data)*

9.2. Supplementary Text 9.2.docx *(validation results)*

9.3. Supplementary Text 9.3.docx *(qRTPCR primers)*

10. Mutational Profiling

10.1. Supplementary Table 10.1.xlsx *(cuffdiff de genes)*

10.2. Supplementary Table 10.2.xlsx *(de pseudogenes)*

10.3. Supplementary Table 10.3.xlsx *(associations)*

10.4. Supplementary Table 10.4.xlsx (intersect fc 3)

10.5. Supplementary Table 10.5.xlsx *(samples)*
