# Supplementary figures and images for: Ensemble Modeling Approach Targeting Heterogeneous RNA-Seq data: Application to Melanoma Pseudogenes
Source: Sci Rep. 2017 Dec 11;7:17344. doi: 10.1038/s41598-017-17337-7 (PMC5725464; doi:10.1038/s41598-017-17337-7)

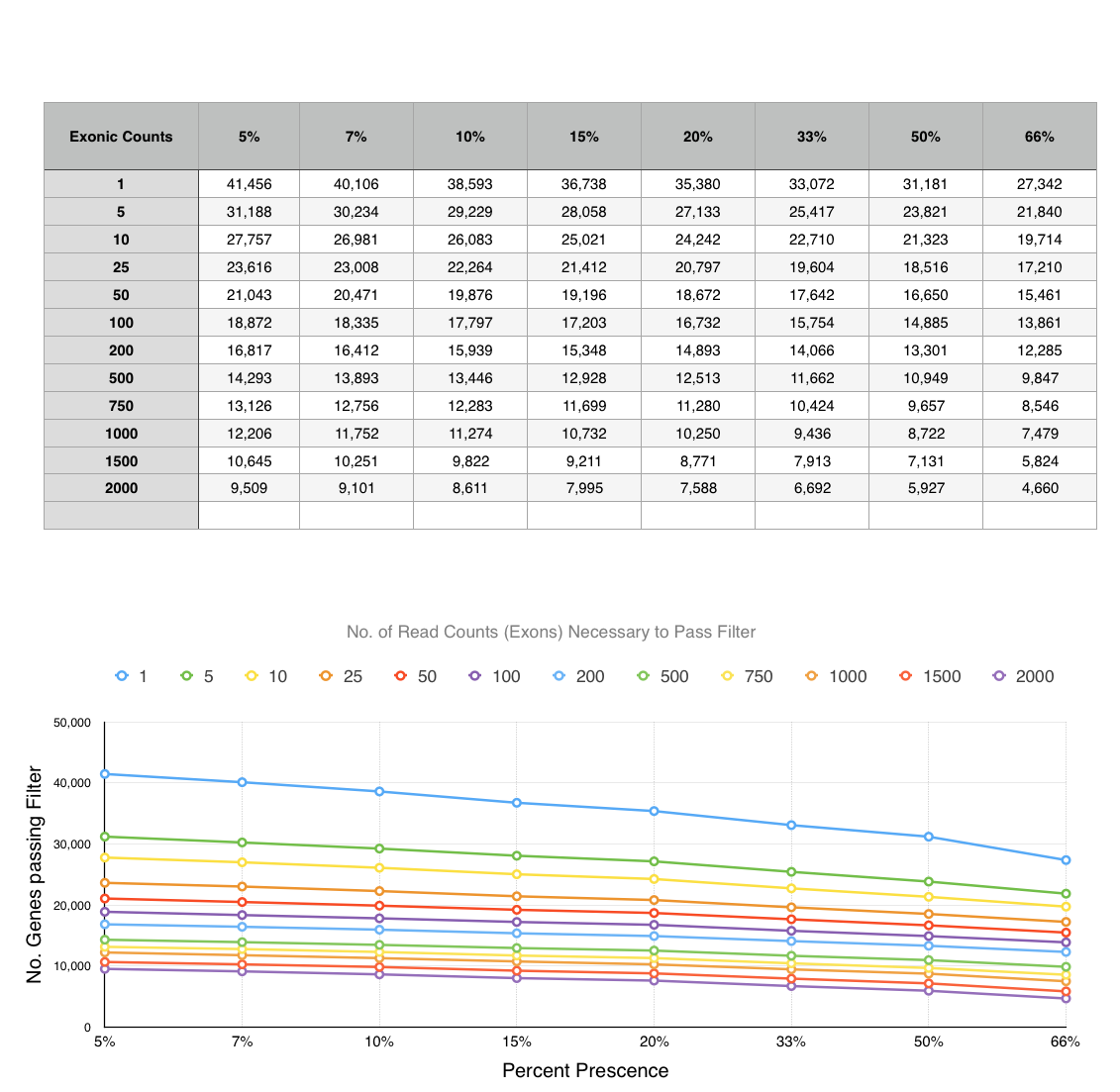

Supplement: Supplementary file 1 — Supplementary Files [file 41598_2017_17337_MOESM1_ESM.zip › Supplementary_Files/4_Sample_Driven_Gene_Detection_Sensitivity_Analysis/Supplementary Fig. 4.1.png]

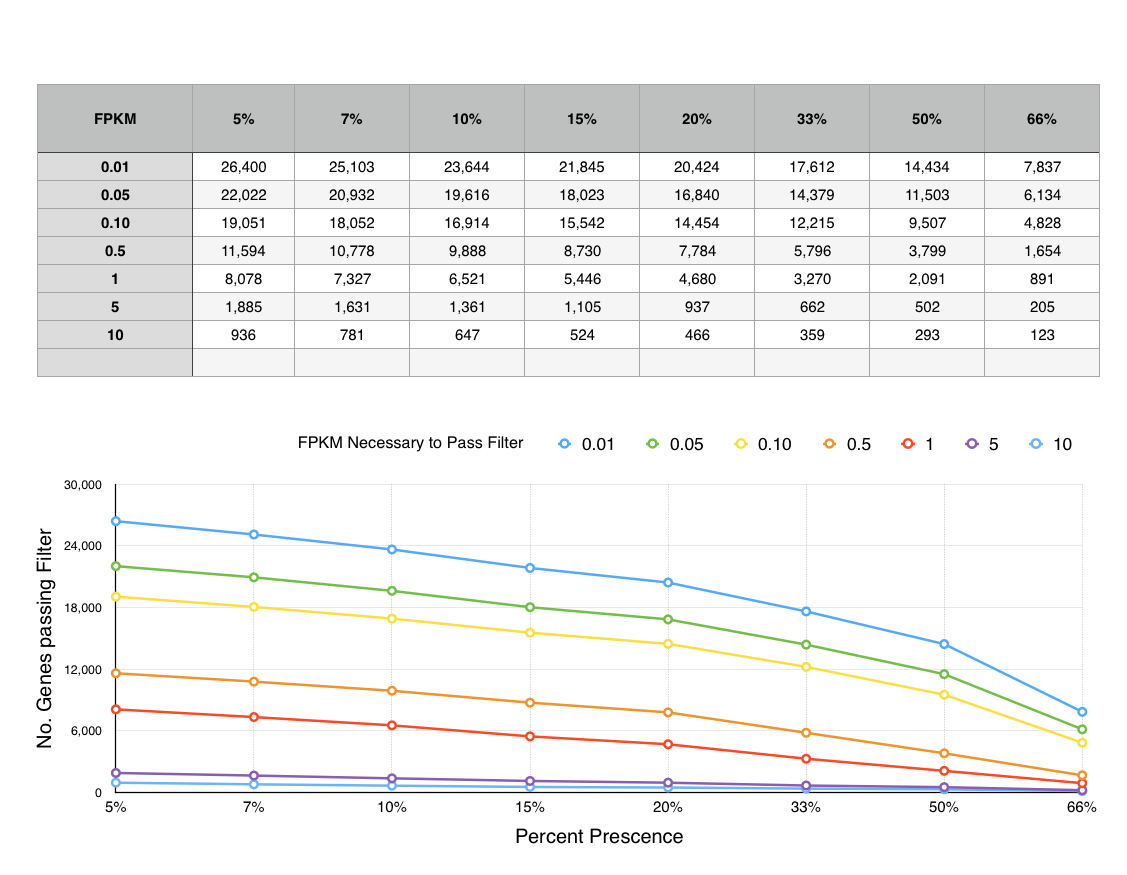

Supplement: Supplementary file 1 — Supplementary Files [file 41598_2017_17337_MOESM1_ESM.zip › Supplementary_Files/4_Sample_Driven_Gene_Detection_Sensitivity_Analysis/Supplementary Fig. 4.2.png]

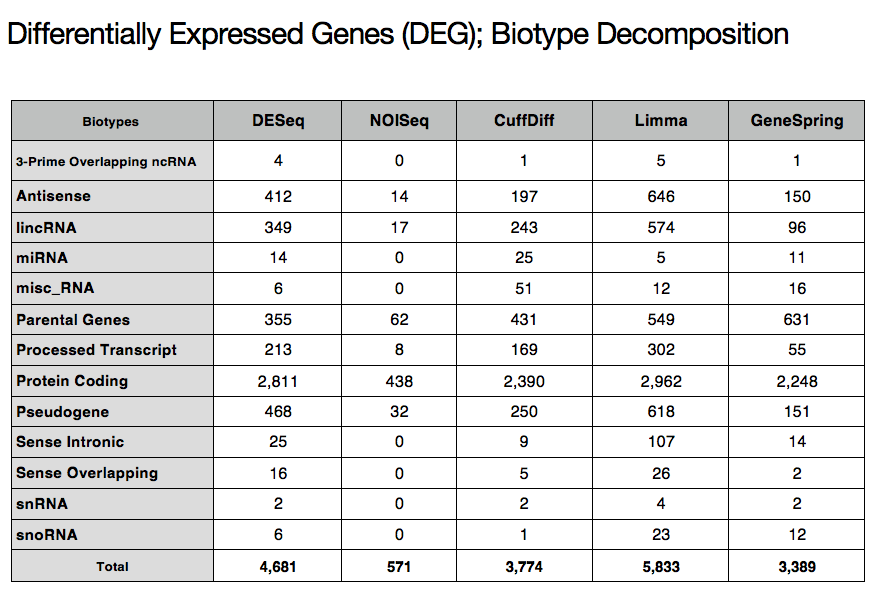

Supplement: Supplementary file 1 — Supplementary Files [file 41598_2017_17337_MOESM1_ESM.zip › Supplementary_Files/5_Cross_Evidences_from_Expression_Profiling/5_2_Biotype_Decomposition/Supplementary Fig. 5.2.1.png]

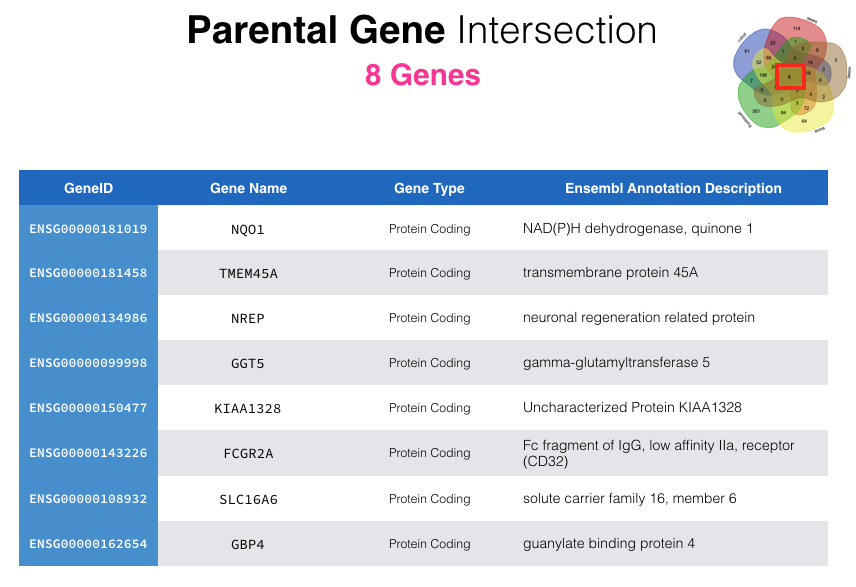

Supplement: Supplementary file 1 — Supplementary Files [file 41598_2017_17337_MOESM1_ESM.zip › Supplementary_Files/5_Cross_Evidences_from_Expression_Profiling/5_3_Consensus_Parental_Gene_Pseudogene_Associations/Suppl Fig. 5.3.3.png]

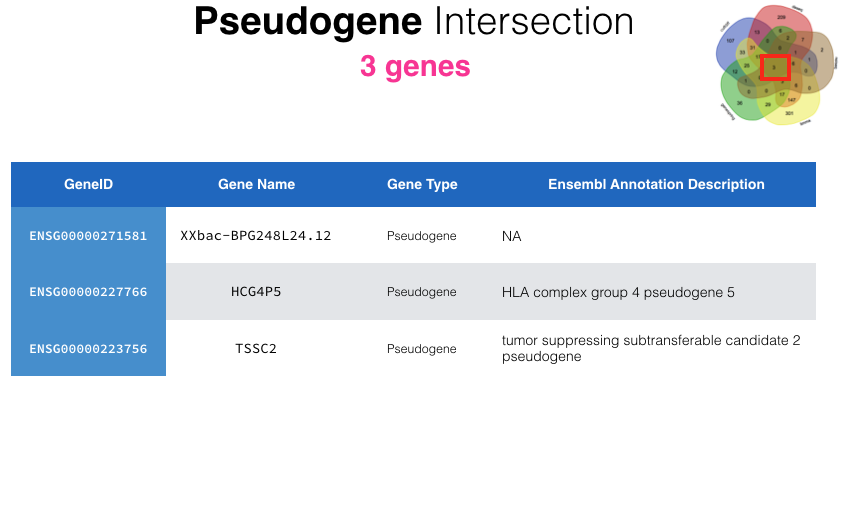

Supplement: Supplementary file 1 — Supplementary Files [file 41598_2017_17337_MOESM1_ESM.zip › Supplementary_Files/5_Cross_Evidences_from_Expression_Profiling/5_3_Consensus_Parental_Gene_Pseudogene_Associations/Suppl Fig. 5.3.4.png]

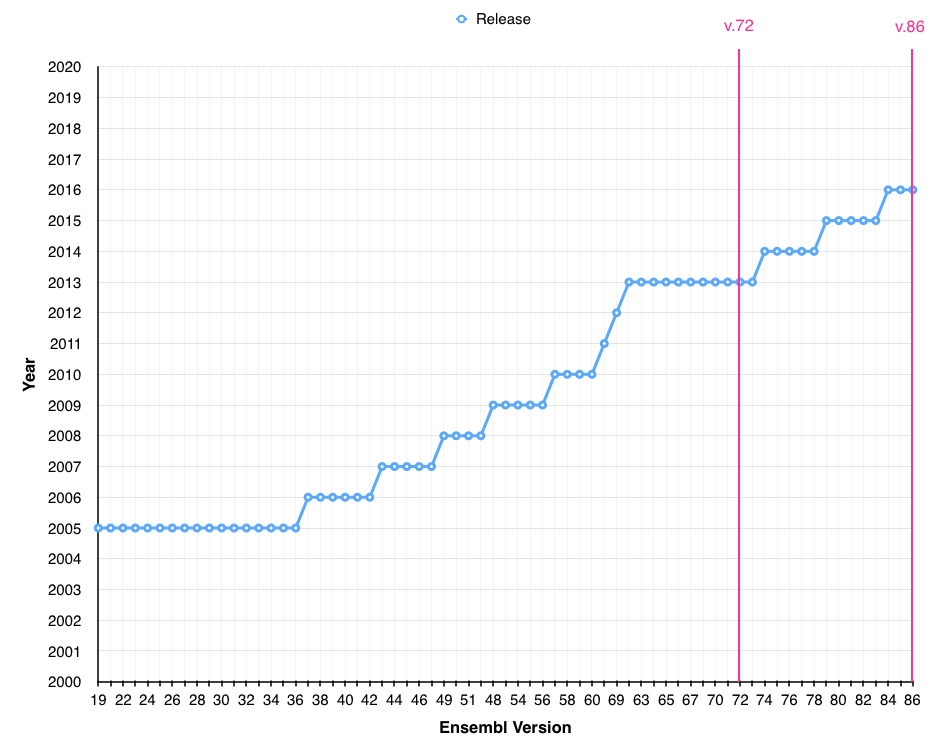

Supplement: Supplementary file 1 — Supplementary Files [file 41598_2017_17337_MOESM1_ESM.zip › Supplementary_Files/6_Bio_Annotations/6_2_Ensembl_Annotations_Comparison/Supplementary Fig. 6.2.4.png]

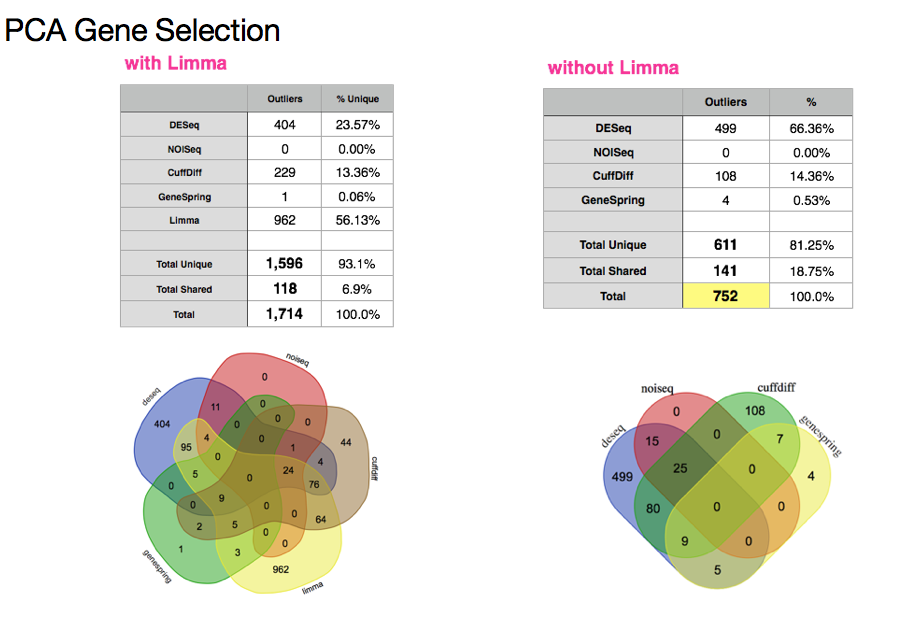

Supplement: Supplementary file 1 — Supplementary Files [file 41598_2017_17337_MOESM1_ESM.zip › Supplementary_Files/8_Models/8_1_PCA_Gene_Selection/Supplementary Fig. 8.1.1.png]

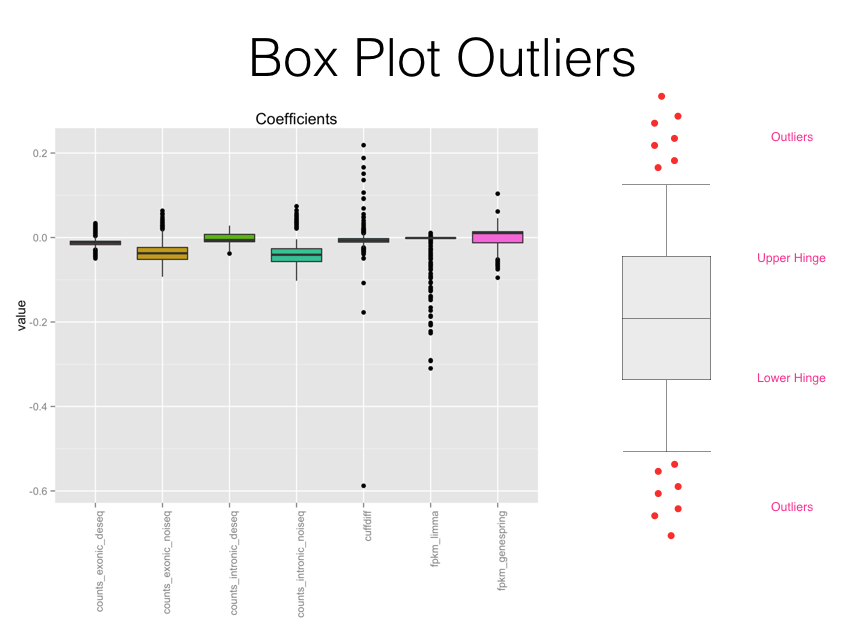

Supplement: Supplementary file 1 — Supplementary Files [file 41598_2017_17337_MOESM1_ESM.zip › Supplementary_Files/8_Models/8_1_PCA_Gene_Selection/Supplementary Fig. 8.1.2.png]

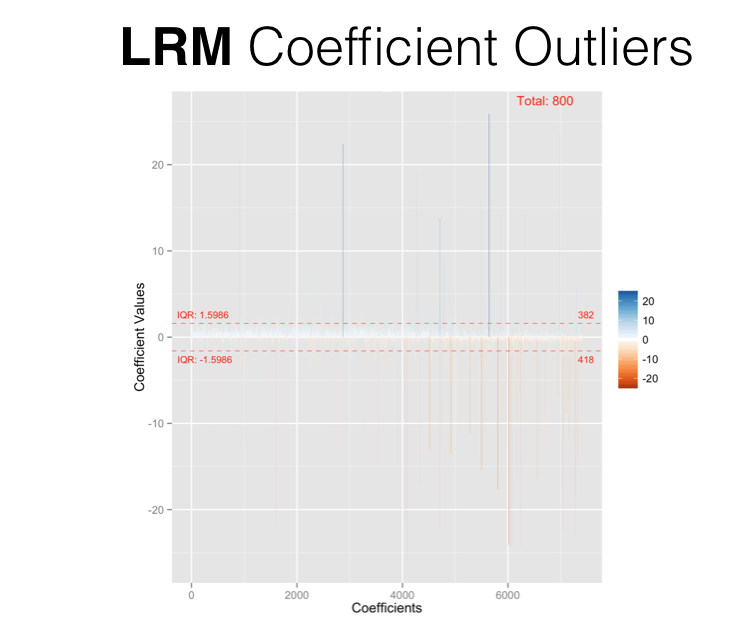

Supplement: Supplementary file 1 — Supplementary Files [file 41598_2017_17337_MOESM1_ESM.zip › Supplementary_Files/8_Models/8_2_Linear_Regression_Model_LRM_Selection/Supplementary Fig. 8.2.10.png]

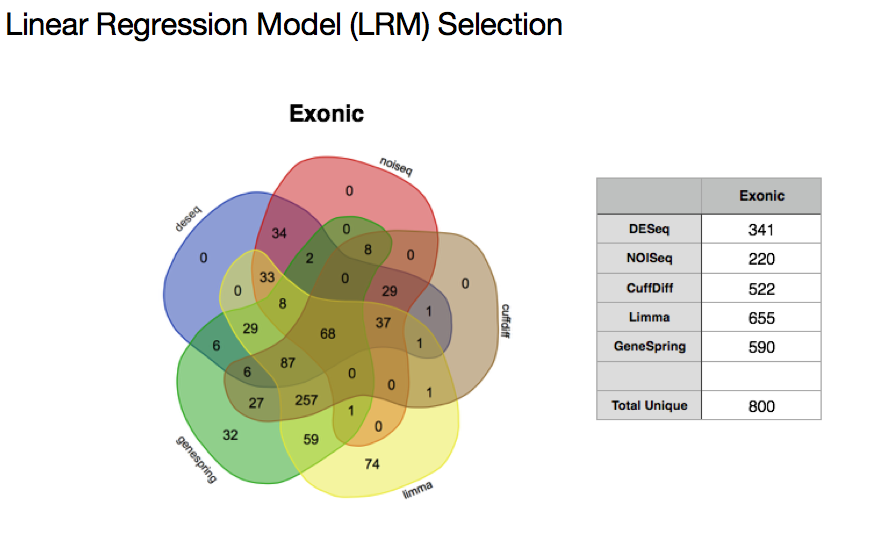

Supplement: Supplementary file 1 — Supplementary Files [file 41598_2017_17337_MOESM1_ESM.zip › Supplementary_Files/8_Models/8_2_Linear_Regression_Model_LRM_Selection/Supplementary Fig. 8.2.9.png]

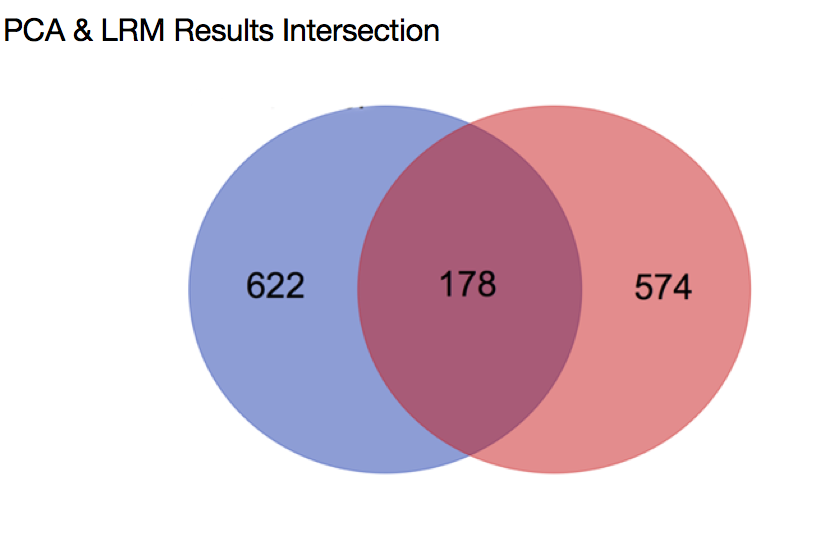

Supplement: Supplementary file 1 — Supplementary Files [file 41598_2017_17337_MOESM1_ESM.zip › Supplementary_Files/8_Models/8_3_LRM_PCA_Results_Intersection/Supplementary Fig. 8.3.1.png]

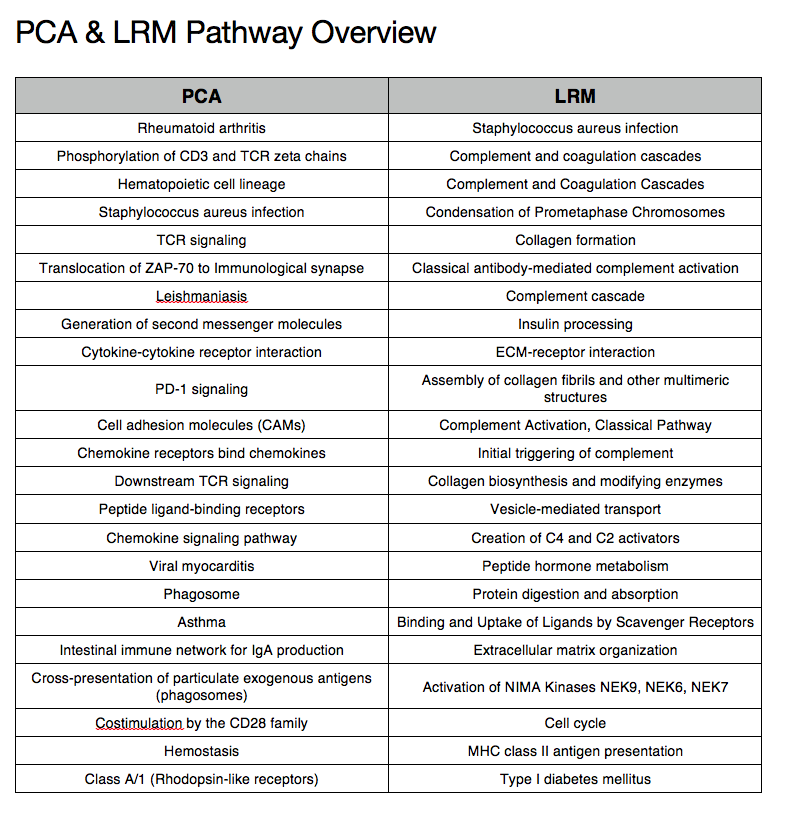

Supplement: Supplementary file 1 — Supplementary Files [file 41598_2017_17337_MOESM1_ESM.zip › Supplementary_Files/8_Models/8_4_LRM_Pathways/Supplementary Fig. 8.4.1.png]

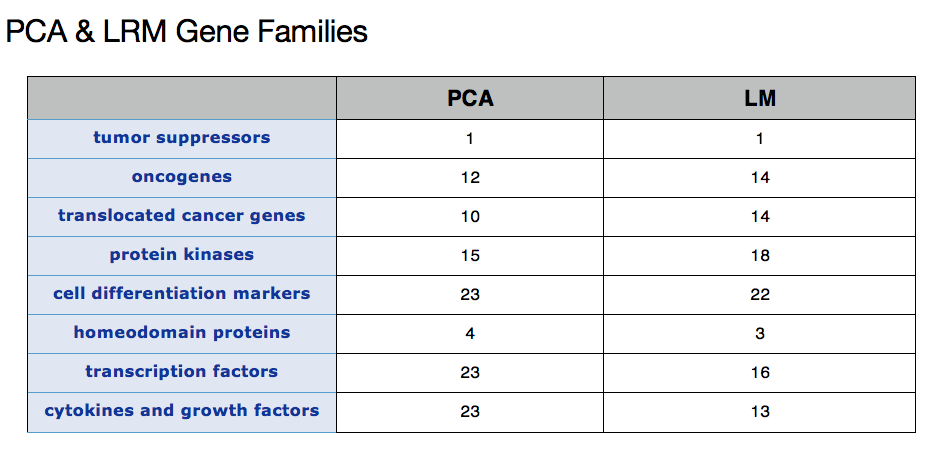

Supplement: Supplementary file 1 — Supplementary Files [file 41598_2017_17337_MOESM1_ESM.zip › Supplementary_Files/8_Models/8_5_LRM_PCA_Gene_Families/Supplementary Fig. 8.5.1.png]

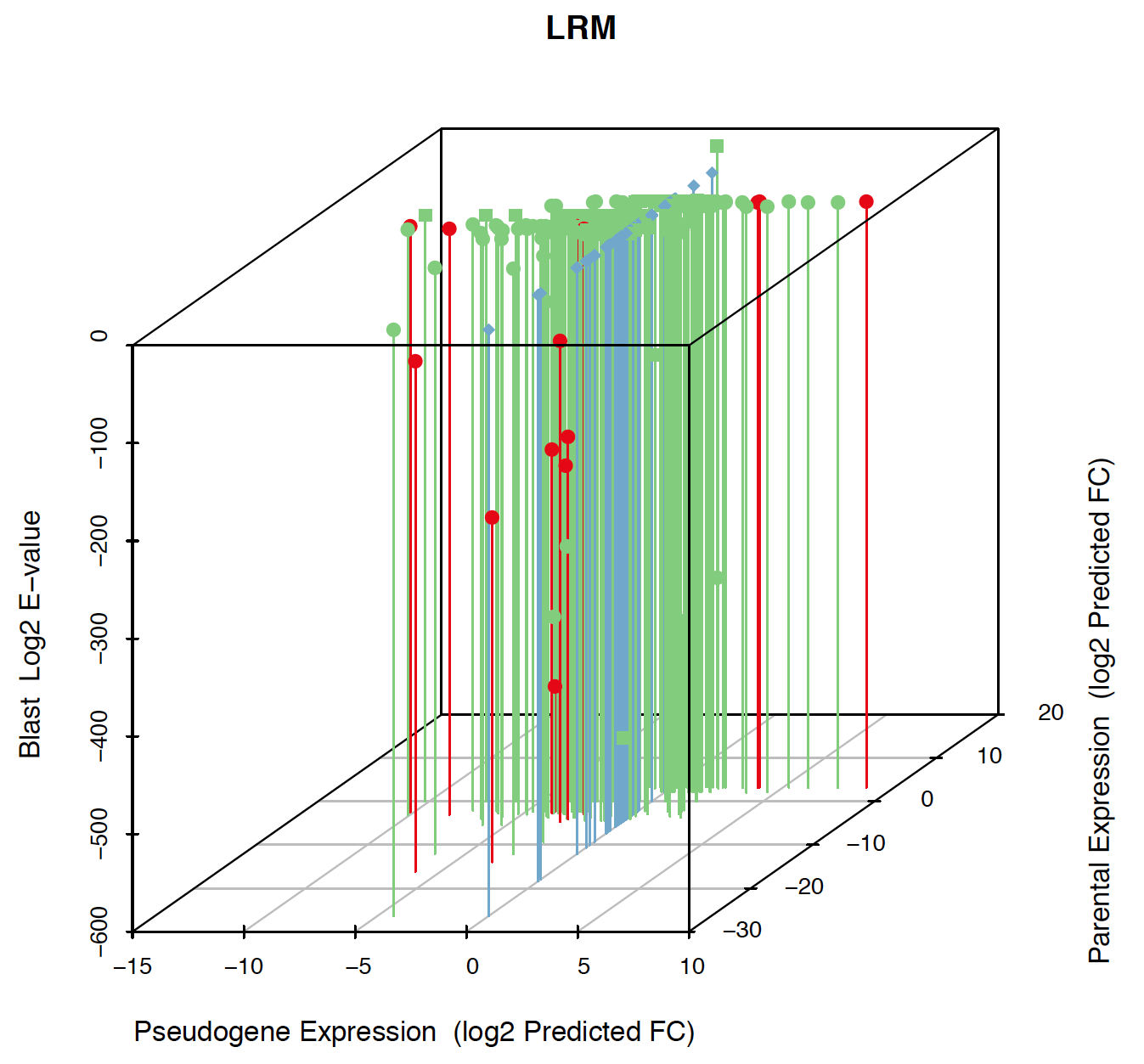

Supplement: Supplementary file 1 — Supplementary Files [file 41598_2017_17337_MOESM1_ESM.zip › Supplementary_Files/8_Models/8_6_Parental_Gene_Pseudogene_Associations/Supplementary Fig. 8.6.1.png]

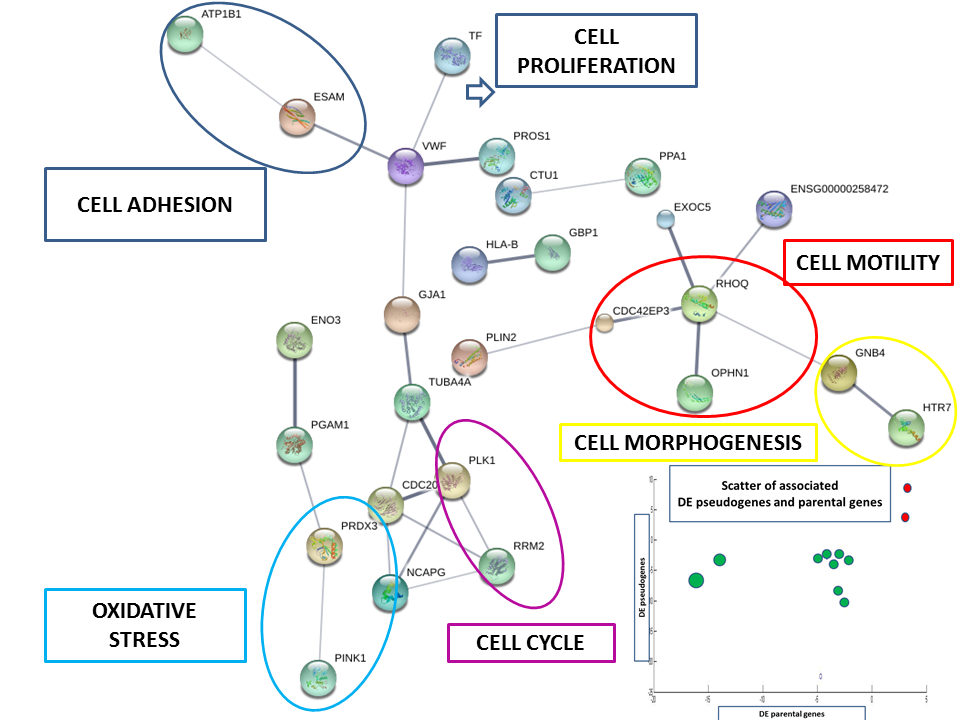

Supplement: Supplementary file 1 — Supplementary Files [file 41598_2017_17337_MOESM1_ESM.zip › Supplementary_Files/8_Models/8_6_Parental_Gene_Pseudogene_Associations/Supplementary Fig. 8.6.2.png]

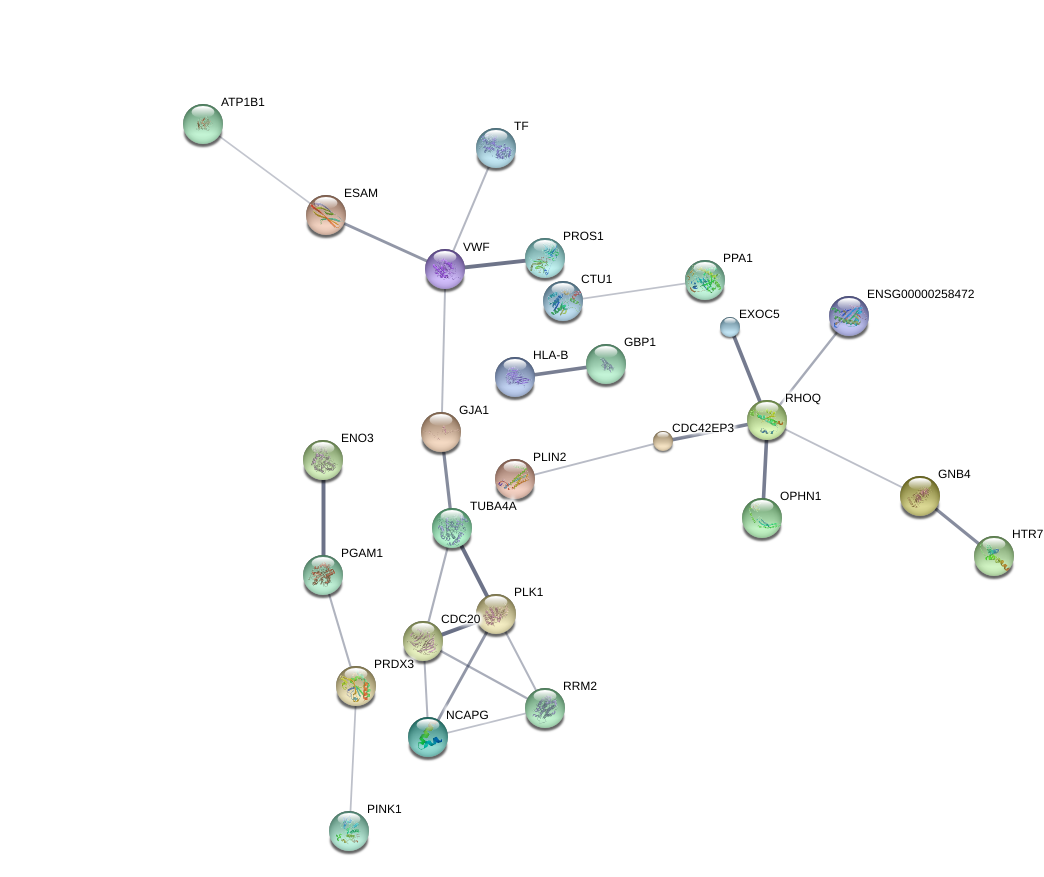

Supplement: Supplementary file 1 — Supplementary Files [file 41598_2017_17337_MOESM1_ESM.zip › Supplementary_Files/8_Models/8_6_Parental_Gene_Pseudogene_Associations/Supplementary Fig. 8.6.3.png]

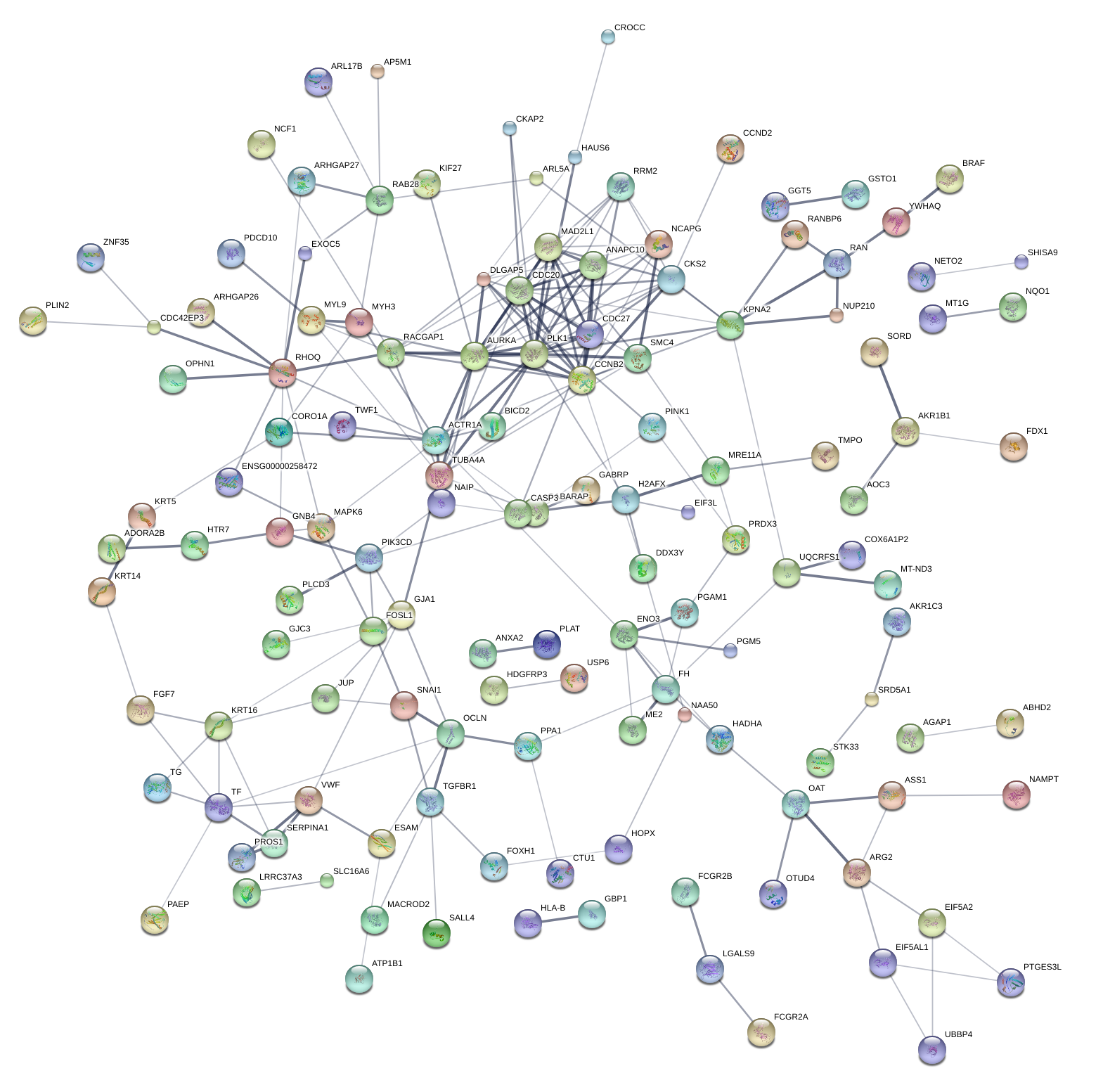

Supplement: Supplementary file 1 — Supplementary Files [file 41598_2017_17337_MOESM1_ESM.zip › Supplementary_Files/8_Models/8_6_Parental_Gene_Pseudogene_Associations/Supplementary Fig. 8.6.4.png]
